# Floral dynamics in the protandrous flowers of *Bauhinia variegata* (Fabaceae): phase-specific morphology, scent, and nectar production

**DOI:** 10.1007/s00425-026-05146-0

**Published:** 2026-09-19

**Authors:** Andrews Vinicius Santos da Silva, Vidal de Freitas Mansano, Dalton Guimarães Veloso, Ana Claudia Fernandes Amaral, Jefferson Diocesano da Cruz, Fabrício de Oliveira Silva, Walter Sotto Mayor Fernandes Neto, Jefferson Rocha de Andrade Silva, Pedro Joaquim Bergamo, Leandro Freitas, Juliana Villela Paulino

**Affiliations:** 1 Programa de Pós-Graduação em Botânica, Escola Nacional de Botânica Tropical, Instituto de Pesquisas Jardim Botânico do Rio de Janeiro, Rua Pacheco Leão 2040, Rio de Janeiro, RJ 22460-036 Brazil; 2 Instituto de Pesquisas Jardim Botânico do Rio de Janeiro, DIPEQ, Rua Pacheco Leão 915, Rio de Janeiro, RJ 22460-030 Brazil; 3 Faculdade de Farmácia, Programa de Pós-Graduação em Ciências Farmacêuticas, Centro de Ciências da Saúde, Universidade Federal do Rio de Janeiro (UFRJ), Rio de Janeiro, RJ 21949-900 Brazil; 4 Laboratório de Plantas Medicinais e Derivados, Farmanguinhos, Fundação Oswaldo Cruz, Rio de Janeiro, RJ 21041-250 Brazil; 5 Departamento de Produtos Naturais e Alimentos, Faculdade de Farmácia, Centro de Ciências da Saúde, Universidade Federal do Rio de Janeiro (UFRJ), Rio de Janeiro, RJ 21941-902 Brazil; 6 Departamento de Química, Laboratório de Cromatografia, Instituto de Ciências Exatas, Universidade Federal do Amazonas (UFAM), Manaus, AM 69077-000 Brazil; 7 Instituto de Biociências, Universidade Estadual Paulista (UNESP), Câmpus de Rio Claro, Avenida 24 A, Rio Claro, SP 151513506-900 Brazil; 8 Instituto Nacional de Ciência E Tecnologia Em Polinização (INPol), Instituto de Pesquisas Jardim Botânico do Rio de Janeiro, DIPEQ, Rua Pacheco Leão 915, Rio de Janeiro, RJ 22460-030 Brazil

**Keywords:** Dichogamy, Floral scent, Molecular docking, Nectar dynamics, Protandry, Volatile organic compounds

## Abstract

**Main conclusion:**

**Protandry in Bauhinia variegata is expressed through coordinated changes in floral morphology, scent composition, secretory activity, and nectar presentation, whereas petal reflectance remains stable; molecular docking further indicates that key phase-associated volatiles are structurally compatible with a bee odourant-binding protein, supporting mechanistic plausibility of olfactory detectability without demonstrating behavioral attraction.**

**Abstract:**

*Bauhinia variegata* exhibits protandrous flowers in which floral morphology, scent production, and nectar presentation change through anthesis. Here, we examined anthesis, stigma receptivity, petal reflectance, volatile organic compound (VOC) composition across floral organs and phases, secretory structures, nectar dynamics, and the structural compatibility of selected phase-discriminant VOCs with a honeybee odourant-binding protein. Flowers exhibited prolonged anthesis, opening at night and remaining functional for 2–3 days. Pollen release predominated during the 1st day (staminate phase), whereas maximal stigma receptivity occurred on the 2nd day (pistillate phase), accompanied by elongation and curvature of the style. Petal reflectance remained stable across phases. Floral scent was spatially heterogeneous among organs and temporally dynamic across anthesis, with terpene-rich bouquets dominated by α-pinene and β-pinene and with phase- or organ-informative compounds including nonanal, limonene, trans-caryophyllene, and 6-methyl-5-hepten-2-one. Cavitated secretory trichomes and sepals showed evidence of terpene-associated secretion. Nectar volume and total sugar content peaked during the pistillate phase, coinciding with strongest stigma receptivity. Molecular docking with AmelOBP14 showed that key phase-associated VOCs are structurally compatible with a bee odourant-binding protein, providing complementary mechanistic support for their potential olfactory detectability. Together, these results show that protandry in *B. variegata* is expressed through coordinated changes in morphology, scent, secretory activity, and nectar presentation, indicating a complex signalling strategy; however behavioural responses of floral visitors remain to be tested.

**Supplementary Information:**

The online version contains supplementary material available at https://doi.org/10.1007/s00425-026-05146-0.

## Introduction

Pollination is a central process in plant sexual reproduction and sustains biodiversity and ecosystem functioning (Elle et al. 2012; Neuschulz et al. 2016). Disruptions in plant–pollinator interactions can reduce reproductive success and may ultimately compromise population persistence, particularly under ongoing habitat loss, biological invasions, and climate change (Harris and Johnson 2004; Segal et al. 2006; Pimm and Raven 2017). Understanding how flowers coordinate signalling and reward presentation over time is therefore essential for interpreting plant reproductive strategies and for guiding effective conservation and regeneration efforts (Neuschulz et al. 2016).

Among angiosperms, many monoecious species temporally separate male and female function through dichogamy, thereby potentially promoting outcrossing (Lloyd and Webb 1986; Brunet and Charlesworth 1995; Aizen 2001; Subramaniam and Bartlett 2023). The staminate phase involves pollen production and dispersal for fertilization while the pistillate phase facilitates pollen reception and seed development. In protandrous species, the staminate phase precedes the pistillate phase, and the duration of floral longevity determines how long each function is displayed and how floral traits may be reconfigured between phases (Pellmyr 1986, 1987; Brunet 1996). Documenting anthesis, stigma receptivity, floral longevity, and coordinated trait shift across distinct phases is therefore fundamental for understanding how dichogamous flowers organize reproduction through time.

Animal-pollinated flowers are multimodal structures in which visual, structural, olfactory, and reward-related traits can change across floral development (Burkle and Runyon 2019; Koski 2020; Larsson et al. 2021). Floral volatile organic compounds (VOCs) are especially relevant and adds further complexity because they are chemically diverse, may vary among organs and across anthesis, and can mediate interactions not only with pollinators but also with florivores, antagonists, and other members of the floral biotic environment (Raguso 2009; Raguso and Weiss 2015; Knudsen et al. 2006; Dötterl and Gershenzon 2023). The same VOC may simultaneously attract pollinators and repel florivores; the ecological outcome of any given compound thus depends on both its chemical identity and the nature of its receiver, which directly limits what can be inferred from phase-specific scent data in the absence of behavioural evidence (Sasidharan et al. 2023). Scent production is also often spatially heterogeneous within flowers, reflecting the contribution of multiple organs and specialized secretory structures, including osmophores and glandular trichomes (Fahn 1988; Solís-Montero et al. 2018; García et al. 2021; Basso-Alves et al. 2022). For this reason, phase-specific and organ-specific scent variation should not be interpreted a priori as direct evidence of “attractiveness”, but rather as part of a dynamic floral signalling phenotype potentially involved in shaping pollinator responses.

Floral rewards are another major component of this phenotype. Nectar secretion and pollen presentation may vary over anthesis according to floral anthesis phase, and environmental conditions, thereby affecting how reproductive investment is distributed through time (Primack 1985; Cresswell 1999; Pacini et al. 2003; Zych et al. 2013). In dichogamous flowers, this temporal structuring of rewards is particularly relevant because staminate and pistillate functions are sequential rather than simultaneous. Thus, phase-dependent nectar and pollen presentation should be interpreted as part of reproductive scheduling and function, even when direct behavioural tests are not available (Charlesworth and Charlesworth 1979; Harder and Barrett 1992; Denisow 2003, 2009, 2011; Denisow et al. 2018).

*Bauhinia variegata* L., an ecologically and culturally significant species that relies heavily on biotic pollinators, is well suited to such an approach. The species bears monoclinous, zygomorphic flowers with pollen and nectar rewards (Raju et al. 2008), and shows evidence of protandry, as in its congeners *B. purpurea* and *B. forficata* (Neto 2013; Rao 2020). In addition, *Bauhinia* sensu stricto presents cavitated secretory trichomes that have been proposed as a distinctive morphological feature of the clade (Duarte-Almeida et al. 2015; Marinho et al. 2016). In *B. variegata*, these structures occur on floral organs directly involved in reproductive display, suggesting that this species offers an appropriate model for integrating floral morphology, anatomy of secretory structures, volatile production, and reward presentation across protandrous phases.

Despite the widespread occurrence of dichogamy among angiosperms, the dynamics of floral signalling and resource availability remain insufficiently explored. Although the inferential limits of descriptive floral datasets must be made explicit (Raguso 2009; Dötterl and Gershenzon 2023), particularly because the emission of a volatile compound during a given floral phase does not, by itself, establish that it attracts pollinators, studies with honeybees have shown that some floral aldehydes, including nonanal, can elicit strong electrophysiological responses in bee antennae without necessarily inducing the strongest positive behavioural responses. In melon flower volatiles, nonanal triggered a marked EAG response in *Apis mellifera*, but the compounds with the highest behavioural attraction in Y-tube olfactometer tests were e−2-hexenal and e−2-octenal (Zhang et al. 2022). Electrophysiological responses of *A. cerana* and *A. mellifera* to pear flower volatiles likewise encompassed a range of aldehydes and terpenes, without uniform behavioural specificity across compounds (Ma et al. 2021). However, in the absence of behavioural or field-choice assays, floral VOCs should be treated as candidate signals whose ecological relevance must be inferred cautiously and, where possible, supported by complementary mechanistic evidence (Raguso 2009; Dötterl and Gershenzon 2023).

Insect odourant-binding proteins (OBPs) provide one such mechanistic point of comparison. These small, soluble extracellular proteins are involved in the peripheral transport of hydrophobic odourant molecules across the aqueous sensillum lymph to olfactory receptors in the antennal dendrites, and their crystallographic structures provide well-defined molecular targets for computational prediction of ligand compatibility (Spinelli et al. 2012; Pelosi et al. 2018; Abendroth et al. 2023). Recent experimental evidence confirmed that AmelOBP2 of *A. mellifera ligustica* participates in the recognition of floral volatiles, particularly aldehydes. After RNAi silencing of AmelOBP2, the electroantennogram response of forager worker bees to 1-nonanal decreased markedly, supporting a functional role of this OBP in the peripheral processing of aldehyde-rich floral signals (Zhang et al. 2025). Within the framework of reverse chemical ecology, molecular docking can therefore be used to evaluate whether phase-discriminant floral VOCs identified by GC–MS display structural compatibility with a well-characterized bee olfactory carrier (Jayanthi et al. 2014; Pelosi et al. 2018). This does not substitute for electrophysiological or behavioural validation but provides a mechanistic rationale for prioritizing compounds for future assays.

We tested the hypothesis that protandry in *B. variegata* is accompanied by coordinated, phase-specific changes in floral morphology, volatile emission, and nectar presentation. To address this hypothesis, we (i) documented anthesis, floral longevity, stigma receptivity, and morphological transitions across the staminate and pistillate phases; (ii) characterized the distribution of scent-associated secretory structures and the spatial and temporal variation of floral VOCs among organs and across anthesis; (iii) quantified phase-dependent nectar production; (iv) assessed phase-dependent pollen availability; and (v) evaluated, through molecular docking, whether key phase-discriminant VOCs show plausible binding compatibility with a structurally characterized honeybee OBP, an approach interpreted here as mechanistic context for peripheral olfactory plausibility, not as evidence of pollinator attraction or behavioural preference.

## Materials and methods

### Study species and collection site of the protandrous species

*Bauhinia variegata* L., commonly known as “Kachnar,” which means “a beautiful glowing lady” in Sanskrit (Kumar et al. 2020), is a medium-sized ornamental tree with large, fragrant flowers, native to Southeast Asia (Zahid et al. 2023). It is widely distributed across tropical and subtropical regions and is pollinated by bees and passerine birds (Raju et al. 2008; Gu et al. 2020).

**Fig. 1:**
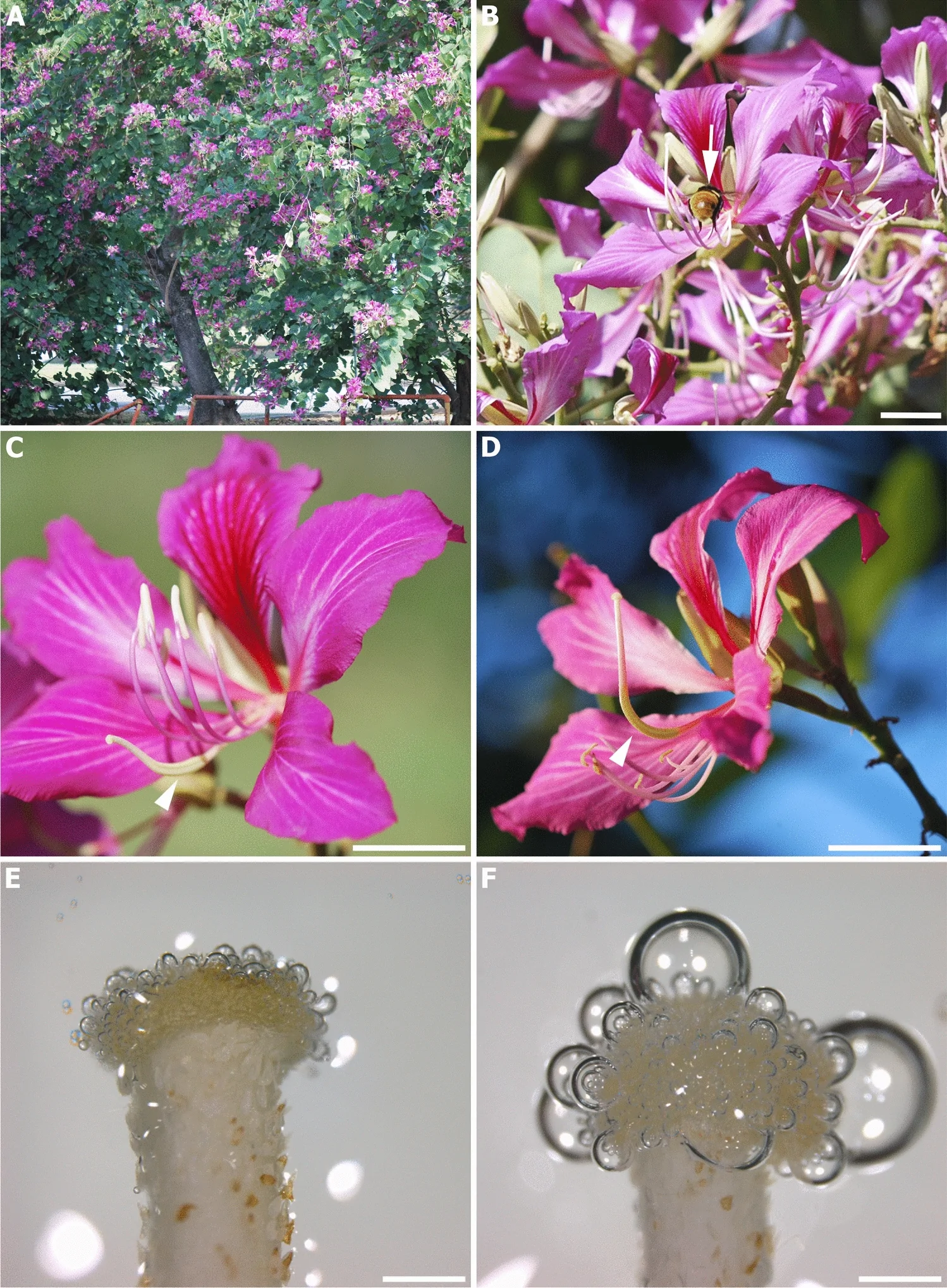
Cultivated individual in collection site: flowers and stigmatic receptivity of *Bauhinia variegata*. **A** Cultivated individual showing inflorescences branches in the collection site. **B** Inflorescence branches being visited by a bee, indicated by a white arrow. **C** Flower on the 1 st day of anthesis (staminate phase). Note the size and curvature of the carpel, indicated by the arrowhead. **D** 2nd day of anthesis (pistillate phase). Carpel length and curvature are very distinguished, indicated by an arrowhead. **E–F** Stigmatic receptivity. **E** Staminate phase carpel with immature stigma exhibiting fewer, smaller oxygen bubbles. **F** Pistillate phase carpel with mature stigma exhibiting abundant, larger oxygen bubbles, indicating peak receptivity. Scale bars: **B**–**D** = 4 cm; **E**–**F** = 1 cm

For the distinct analyses of floral traits and rewards, plant material, including flowers and floral buds, was collected from adult, reproductively mature cultivated individuals of *B. variegata* during both the staminate phase (SP) and pistillate phase (PP) of flowering. Collections were made from 2021 to 2024 at the Living Plant Collection of the Faculty of Letters, Federal University of Rio de Janeiro, Brazil, South America (Fig. 1A-B; 22° 51′ 36.797" S 43° 13′ 30.810" W). A voucher specimen (RB 877065) has been deposited in the RB Herbarium.

Ten pre-anthesis flowers were selected from different branches to ensure representative coverage of the tree canopy and were marked in June 2021. Measurements were expressed as mean ± standard deviation (mm) to document the key morphological changes associated with floral opening, anthesis, and senescence.

To document the anthesis dynamics, floral buds were observed hourly during the first night to precisely determine the onset of anthesis. After this initial period, observations continued every 12 h for 3 days ending with the onset of senescence, indicated by petal wilting. Photographs were taken using a Nikon D60 digital camera. *B. variegata* exhibits a nocturnal onset of anthesis, with flowers beginning to open between 7 and 9 PM.

To investigate stigma receptivity, we used the hydrogen peroxide method (Kearns and Inouye 1993), which assesses peroxidase activity in receptive stigmas based on the formation of oxygen bubbles. For this analysis, ten flowers were selected from each floral phase. A 3% hydrogen peroxide solution was applied to stigmas placed on Petri dishes, and the formation and distribution of bubbles were observed for 1 min. The reactions were photographed, and the images were analyzed to compare bubble formation.

### Scanning electron microscopy (SEM)

To examine floral morphology and the surface of cavitated secretory trichomes under SEM, mature flowers were collected and fixed in FAA 70 (formalin:acetic acid:alcohol; Johansen 1940), then gradually dehydrated in ethanol series, and stored in 70% ethanol. Samples were dissected under a Heerbrugg Wild 308,700 (Gais, Switzerland) and examined using Leica Wild MZ8 and Leica SAPO stereoscopic microscopes (Leica Microsystems, Wetzlar, Germany). Then, they were dried using critical point dryers (Quorum K850, Quorum Technologies, Lewes, UK; Leica EM CPD300, Leica Microsystems, Wetzlar, Germany; and Bal-Tec CPD 030, BAL-TEC, Bannockburn, IL, USA). The dried samples were mounted on metal stubs with carbon adhesive tape and sputter-coated with gold using a Quorum Q15OT ES (Quorum Technologies, Lewes, UK), Leica EM SCD 050 (Leica Microsystems, Wetzlar, Germany), and Emitech K550X and K575X (Ashford, United Kingdom). Observations were made using scanning electron microscopes: LEO Supra 55VP (RBGE), Hitachi Regulus 8230 (Kew), and JEOL—JSM-6490LV (CBPF/RJ) operating at 5, 10, or 15 kV. Electron micrographs were processed using Adobe Photoshop CS6 (San Jose, California, USA).

### Petal reflectance

To test whether petal reflectance changes between the staminate and pistillate phases, we measured the floral reflectance of flowers from five individuals of *B. variegata* at the collection site. On the 1 st day of anthesis (staminate phase), one flower per individual (*n* = 5) was selected. On the 2nd day (pistillate phase), a new flower (*n* = 5) was selected from each of the same individuals. In each flower, reflectance was measured on both the lateral petals and the median adaxial petal bearing the nectar guide, as these are the most visually prominent petal regions of the flower.

Reflectance was measured using a USB 4000 surface spectrophotometer (OceanOptics) at a 45° angle. Barium sulfate was used as the white standard, and a dark chamber served as the black standard (Lunau et al. 2011). We calculated the average reflectance for each floral part and anthesis phase to visually compare reflectance curves. Analyses were restricted to the 300–700 nm wavelength region, which corresponds to the visual spectrum of most pollinators (Lunau et al. 2011).

### Detection of scent signalling

#### Histochemical analyses

To determine the specific floral tissues involved in scent production and emission, we used histochemical staining techniques. Fresh flowers at anthesis in both phases (ST and PP) were immersed in a neutral red solution (1:10.000) in situ for 1 h (Vogel 1990) and examined under a stereomicroscope to detect potential scent-production tissues. Neutral red is a vital stain that accumulates in the vacuoles of metabolically active cells, potentially indicating tissues involved in the storage or secretion of volatile compounds.

Subsequently, based on the neutral red testes, fresh sepals and carpels, which bear cavitated secretory trichomes, were hand-sectioned using razor blades and stained with Nadi reagent (David and Carde 1964) to detect essential oils. Unstained control sections were performed and examined. Photomicrographs were obtained using an LDM 750 light microscope (Leica Microsystems, Wetzlar, Germany) equipped with an integrated Leica Flexcam i5 digital camera (Leica Microsystems, Wetzlar, Germany).

#### VOCs collection

To characterize changes in floral scent composition throughout anthesis, we collected VOCs emitted by the flowers. Sixty pre-anthesis floral buds from ten different individuals were isolated at the beginning of the night before anthesis using plastic bags containing a charcoal cartridge to adsorb and trap VOCs. After 24 h of the first flower opened, i.e. in staminate phase (SP), 30 flowers were collected and the cartridges were removed. New cartridges were then inserted into the remaining 30 bagged flowers. After another 24 h, corresponding to the pistillate phase, these flowers were collected, and the cartridges were removed. For the remaining 30 flowers, two additional cartridges were used: one was subsequently added to represent the pistillate phase (PP), and the other remained in place throughout the entire sampling period, representing complete anthesis (CA), from the staminate through the pistillate phase.

VOCs were immediately eluted from the cartridges using 2 mL of HPLC/UV grade dichloromethane (TEDIA, Brazil), a solvent effective in dissolving a wide range of volatile compounds. The resulting extracts were transferred to amber vials, sealed, and stored at 4 °C until analysis by gas chromatography coupled to mass spectrometry (GC–MS).

#### Cavitated secretory trichomes collection

To analyse the chemical composition of secretions from cavitated secretory trichomes and compare the staminate and pistillate phases, trichomes were manually removed using a stereomicroscope and an acupuncture needle from the carpel surface of freshly collected flowers in two distinct time points:1) trichomes from carpels of flowers collected between 8:00 and 10:00 AM on the 1 st day after anthesis (staminate phase), and 2) trichomes from carpels of flowers collected between 8:00 and 10:00 AM on the 2nd day after anthesis (pistillate phase). Trichomes were placed in Eppendorf tubes containing 1 ml of distilled water and were then immediately frozen at −20 °C and stored at this temperature until further processing.

#### Headspace solid-phase microextraction (HS-SPME)

HS-SPME analyses of *B. variegata* floral organs and trichomes from both phases were performed using a 100 µm polydimethylsiloxane fiber conditioned in a 50% water:methanol solution for 30 min and heated in the GC injection port at 260 °C for 10 min. For extraction, we used 250 mg of plant material, 25% NaCl, and 5 mL of Milli-Q water in 20 mL glass vials sealed with aluminium seals and septa. The vials were heated in a water bath at 40 °C with magnetic stirring to facilitate volatile release. After 10 min of equilibration between the solution and the expansion chamber, the fiber was exposed to the headspace for 40 min to adsorb the analytes. After the adsorption time, the fiber was reinserted into the GC injection port to start the chromatographic analysis. All analyses were performed in triplicate.

#### Volatile compounds analysis by GC–MS

VOCs were analysed using a Shimadzu GC-2010 plus gas chromatograph coupled with a QP-2010 Mass Selective Detector (ionization voltage 70 eV). The system was equipped with a nonpolar DB-5MS capillary column (30 m × 0.25 mm, 0.25 μm film thickness), and helium was used as the carrier gas at a flow rate of 1.0 mL/min. The oven temperature was programmed to increase from 50 °C to 260 °C at 7 °C/min, followed by a 5-min isothermal hold at 260 °C. Injector and detector temperatures were maintained at 250 °C. The linear velocity was set to 14 cm/s. The MS interface temperature was 280 °C, mass spectra were recorded over a mass range of 40–700 Da, with a scan speed of 150 u/s and a scan interval of 0.50 s (2 Hz). All analyses were conducted in triplicate. Volatile compounds were identified by matching their experimental retention behaviour and mass spectral fragmentation patterns against published retention indices and authenticated mass spectra available in peer-reviewed literature and the Wiley spectral library.

#### Resource: nectar measurement

To investigate the dynamics of nectar production and availability throughout anthesis, pre-anthesis floral buds were bagged to prevent pollinator access. Nectar volume and sugar concentration were determined at four time points after anthesis: 12 and 24 h, corresponding to the staminate phase (*n* = 26 and *n* = 16, respectively), and 36 and 48 h, corresponding to the pistillate phase (*n* = 14 and *n* = 7, respectively). Measurements were performed at the collection site. Nectar volume was measured using a 25 µL graduated microsyringe (Hamilton Co., Reno, Nevada, USA; model 702), while sugar concentration was determined using a handheld refractometer (Bellingham + Stanley, model 45–03). Sugar concentration, expressed in °Brix, was converted to sugar mass per unit volume (mg/µL) using the equation: *y* = 0.00226 + (0.00937x) + (0.0000585x^2^), where x corresponds to sugar concentration (%) and y to sugar mass per microliter of nectar (Dafni et al. 2005). Total sugar content per flower (mg) was calculated by multiplying nectar volume (µL) by sugar mass per unit volume (mg/µL) (Table S1).

### In silico analysis

#### Receptor structure and ligand set

The crystallographic structure of *Apis mellifera* odourant-binding protein 14 (AmelOBP14) in complex with citralva (PDB ID: 3S0D; resolution 1.24 Å) was retrieved from the Protein Data Bank and used as the receptor in the docking analyses. This structure was chosen because AmelOBP14 has been characterized in complex with odourant molecules relevant to olfactory recognition (Spinelli et al. 2012). Prior to docking, the co-crystallized ligand was removed from the binding pocket, and the receptor structure was prepared using the default settings of the docking platform.

Isomeric SMILES of the ligands were obtained from the PubChem database (National Center for Biotechnology Information 2026). Two-dimensional structures were generated with ChemDraw Professional 12.0, while three-dimensional structures and file conversions were performed using the Chem3D extension. Energy minimization was carried out with the MM2 force field until convergence was achieved under default settings. The optimized 3D structures were saved in MOL2 format for subsequent docking analyses.

The ligand set included 6-methyl-5-hepten-2-one, α-pinene, β-pinene, nonanal, trans-caryophyllene, β-elemene, eugenol, R-limonene, and S-limonene. Eugenol was incorporated as a positive reference ligand, given its experimentally determined sub-micromolar dissociation constant for AmelOBP14, measured by fluorescence displacement assays (Spinelli et al. 2012; Mayack et al. 2023). Each ligand structure was individually optimized and converted into formats compatible with the docking calculations.

#### Molecular docking

Molecular docking was performed using GOLD v.2024.3.0 (Cambridge Crystallographic Data Centre, CCDC), employing the ChemPLP scoring function (Jones et al. 1997; Verdonk et al. 2003). The binding region was defined based on the ligand-binding cavity occupied by the co-crystallized ligand in the 3S0D structure. Docking runs were carried out using the default genetic algorithm parameters, automatic early termination criteria, and cavity detection enabled. For each ligand, the 20 top-ranked poses were generated, and the resulting complexes were ranked according to ChemPLP fitness scores.

#### Analysis of docking results

For each compound, the pose with the highest ChemPLP fitness value was selected for comparative analysis. The resulting receptor–ligand complexes were inspected to identify the presence of hydrogen bonds and hydrophobic contacts within the AmelOBP14 binding pocket using two-dimensional interaction diagrams. Docking outputs were interpreted comparatively, emphasizing the relative structural compatibility of the tested floral volatiles with the olfactory carrier protein rather than direct behavioural inference. Consistency of pose rankings was assessed by comparing the top three solutions for each ligand; compounds showing limited score variation among top-ranked poses were considered to yield reproducible binding geometries.

#### Molecular dynamics simulation

To assess the dynamic stability of selected docking complexes, molecular dynamics (MD) simulations were performed for four compounds: trans-caryophyllene, selected for detailed analysis because it combined high ChemPLP fitness with zero torsional penalty and broad hydrophobic contact coverage; β-elemene, which yielded the highest docking score overall; nonanal, which increased in petals during the pistillate phase and has independent electrophysiological support in *Apis*; and eugenol, used as a dynamic benchmark because its sub-micromolar dissociation constant for AmelOBP14 has been experimentally determined (Spinelli et al. 2012; Mayack et al. 2023; Zhang et al. 2025). The protein–ligand complexes corresponding to the top-ranked docking poses were used as starting structures. All MD simulations were performed using the Sibiolead platform with GROMACS, employing the CHARMM36 force field for the protein and CGenFF parameters for the ligands, in an explicit TIP3P water box with a minimum solute-to-box edge distance of 1.0 nm and periodic boundary conditions. Systems were energy minimized, equilibrated in NVT (100 ps) and NPT (100 ps) ensembles, and simulated for 100 ns at 310 K and 1 bar. Production trajectories were analysed for backbone RMSD, per-residue RMSF, radius of gyration (Rg), and number of intramolecular hydrogen bonds using GROMACS analysis tools. Comparative plots in the main text focus on the two floral VOCs of strongest biological relevance in the present system, nonanal and trans-caryophyllene, whereas eugenol and β-elemene are retained as text-supported benchmark/supporting analyses summarized in Table S2.

## Statistical analysis

For the floral-organ dataset, each organ was sampled on the 1 st and 2nd day of anthesis; each organ × day combination was analysed in three biological replicates. For the anthesis-phase dataset, three floral samples were collected per phase, each analysed in three instrumental replicates, which were averaged prior to analysis. GC–MS data were processed in MetaboAnalyst 4.0 (Chong et al. 2018). Compounds detected in fewer than 50% of samples were removed and remaining missing values replaced by the minimum detected value of the corresponding variable. The floral-organ dataset was normalized by sample median, log-transformed and mean-centred; the anthesis-phase dataset was normalized by sample median, square-root transformed and Pareto-scaled. PCA, PLS-DA and OPLS-DA were applied, with VIP > 1.0 taken as indicative of discriminatory compounds (Chen et al. 2009). PLS-DA models were assessed by cross-validation (R^2^, Q^2^)—fivefold for the floral-organ dataset and leave one out for the anthesis-phase dataset, given its smaller sample size — and by permutation of class labels using the between-group to within-group sum-of-squares ratio as the test statistic. Group separation in PCA was tested by PERMANOVA on PC1–PC2 scores. Hierarchical clustering used Euclidean distance and Ward’s linkage. Individual compounds were compared among anthesis phases by one-way ANOVA on the averaged biological replicates (*n* = 3 per phase).

Nectar data normality was assessed using the Shapiro–Wilk test. For non-parametric data, the Kruskal–Wallis test was used, followed by Dunn’s test for multiple comparisons among groups. *p*-values ≤ 0.05 were considered statistically significant. Statistical analyses were performed using GraphPad Prism 10 (GraphPad Software, Boston, MA, USA.

## Results

### Floral morphology and dynamic anthesis across the distinct floral phases

The flowers are complete, heterochlamydeous, pedicellate, pentamerous, and zygomorphic. They exhibit distinct floral morphology across the protandrous phases. The monocarpellate gynoecium consists of a superior ovary bearing both non-glandular and glandular trichomes on its surface, an erect, cylindrical style, and a terminal, capitate stigma covered with indumentum. The style undergoes a marked morphological change during floral anthesis.

On the 1 st day of anthesis (staminate phase), the style is relatively short and white, measuring 49.76 ± 5.76 mm in length (Fig. 1C). The stigma remains unreceptive, as detected by the hydrogen peroxide test. Pollen release from the anthers is initiated upon flower opening and continues throughout the 1 st day, with most pollen grains shed within the first 12 h. By the 2nd day (pistillate phase), the style elongates considerably, reaching 66.26 ± 8.59 mm in length, curves distinctly toward the adaxial side of the flower, and develops a pink hue (Fig. 1D). At this stage, the stigma becomes receptive, coinciding with the depletion of pollen grains and the onset of anther senescence, regardless of visitor interactions.

Stigma receptivity tests revealed differences between the staminate and pistillate phases of *B. variegata* L. flowers. During the staminate phase, carpels produced fewer and smaller oxygen bubbles (Fig. 1E) than those observed during the pistillate phase (Fig. 1F), indicating greater stigma receptivity during the latter phase. Floral senescence generally occurs 2–3 days after anthesis.

### Floral reflectance

**Fig. 2:**
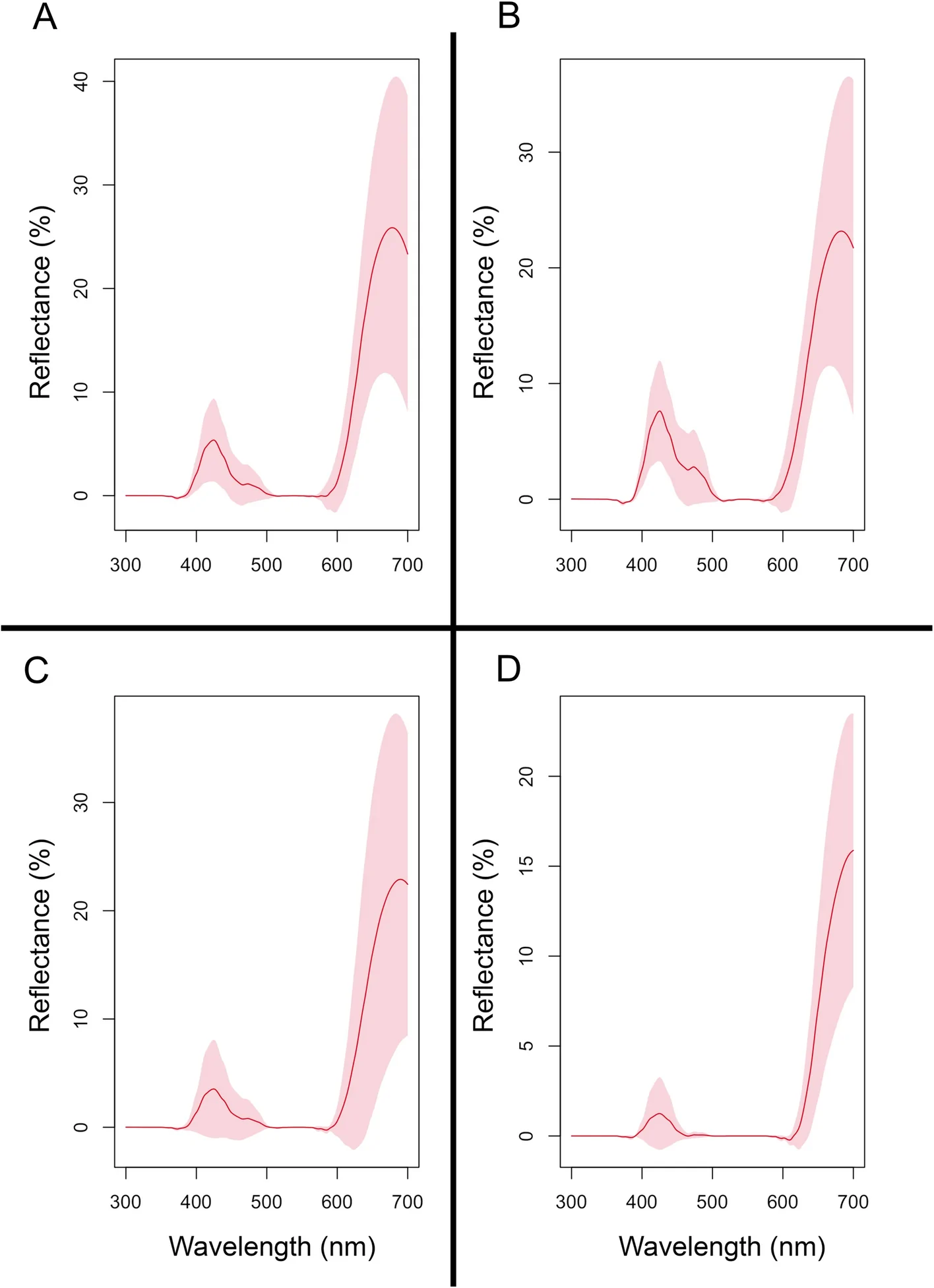
Spectral reflectance of petals of *Bauhinia variegata*. **A-B** Central petal. **C-D** Lateral petal. **A** and **C** correspond to the staminate phase, whereas **B** and **D** correspond to the pistillate phase. Colour bars are individual flowers, and the bold line represents the mean

Both the lateral petals and the adaxial petal that has a nectar guide showed similar reflectance patterns (Fig. 2). A major reflectance peak was observed in the red region (600–700 nm) and a secondary peak in the blue region (400–500 nm), which gives petals the characteristic pink to reddish coloration. No ultraviolet (UV) reflection was observed. Reflectance curves remained similar throughout the anthesis, with no detectable changes in spectral characteristics between the staminate and pistillate phases in both lateral and central petals.

### Floral scent variation among floral organs

**Fig. 3:**
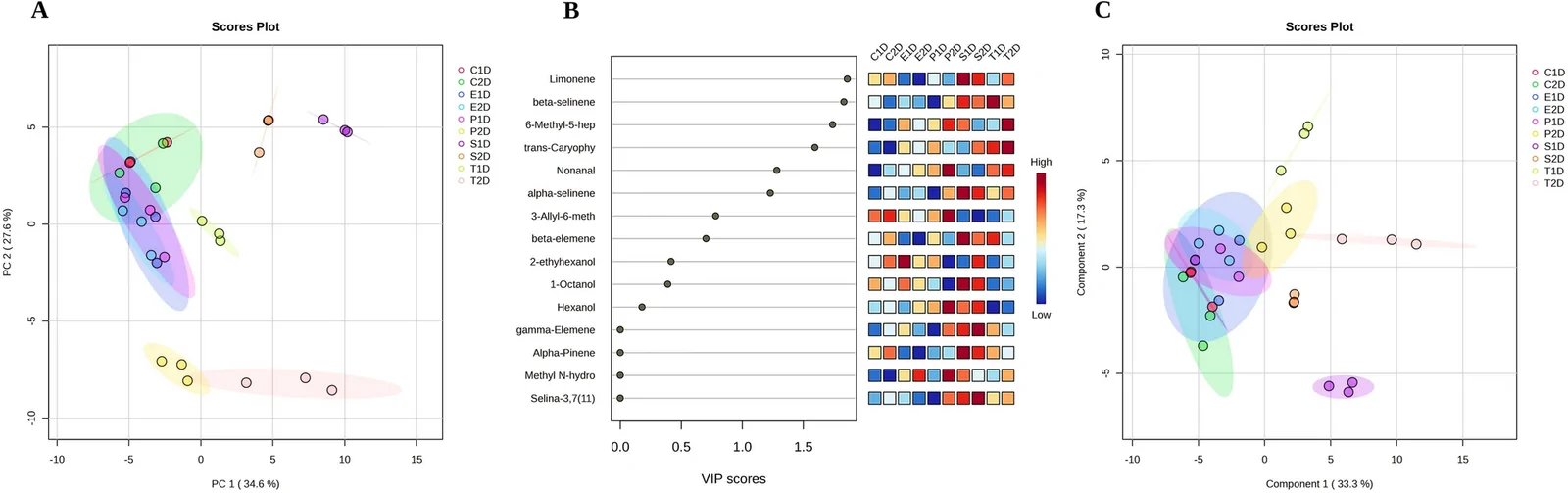
PCA score plot (**A**) (PERMANOVA: *F* = 45.13, R^2^ = 0.953, *p* = 0.001), VIP (**B**) of floral whorls of *Bauhinia variegata*, and PLS-DA score plot (**C**) (four components; R^2^ = 0.895, Q^2^ = 0.793; permutation test *p* < 0.01). *P1D* petals 1 st day, *P2D* petals 2nd day, *C1D* carpels 1 st day, *C2D* carpels 2nd day, *E1D* stamens 1 st day, *E2D* stamens 2nd day, *S1D* sepals 1 st day, *S2D* sepals 2nd day, *T1D* trichomes 1 st day, *T2D* trichomes 2nd day

The floral scent of *B. variegata* is remarkably complex, composed of a diverse array of VOCs from various chemical classes, including alcohols, hydrocarbons, terpenes, and terpenoids, each derived from distinct metabolic pathways (Table S3). The PCA score plot (Fig. 3A) captured 62.2% of the total variation, with PC1 and PC2 accounting for 34.6% and 27.6%, respectively (PERMANOVA: *F* = 45.13, R^2^ = 0.953, *p* = 0.001). A prominent cluster of VOCs emitted by petals on the 1 st day of anthesis, as well as carpels and stamens from both observation days, is concentrated in the upper left quadrant of the plot, with a small dispersion in the lower left quadrant. The main compounds released by petals on the 1 st day were hexadecyl octanoate, γ-elemene, α-pinene, and methyl N-hydroxybenzimidate. In carpels, the dominant VOCs on both observation days were α-pinene (in a concentration nearly three times higher than in petals), β-pinene (higher concentration on the 1 st day and absent in petals and stamens), and γ-elemene. The main VOCs in stamens were hexanol, methyl N-hydroxybenzimidate, γ-elemene, and 3-allyl-6-methoxyphenol. VOCs emitted by sepals on both observation days clustered in the upper right quadrant of the PCA plot and consisted mainly of methyl N-hydroxybenzimidate, β-pinene, and α-pinene, the latter showing the highest concentration among all floral organs analysed. The VOC profile released by petals on the 2nd day of anthesis formed a distinct cluster in the lower left quadrant.

Concerning trichomes, VOCs emitted on the 1 st day occupied a central position on the PCA plot, with a tendency toward the lower right quadrant. On the 2nd day, VOCs formed a separate cluster in a more distant position within the same quadrant. VOC emission by trichomes was considerably lower than that of the floral organs, with γ-elemene identified as the predominant compound.

PLS-DA confirmed the group separation previously observed in PCA (Fig. 3C) (four components; R^2^ = 0.895, Q^2^ = 0.793; permutation test *p* < 0.01) and allowed the identification of compounds contributing to this separation based on the VIP scores (Fig. 3B). Limonene, α-selinene, β-selinene, 6-methyl-5-hepten-2-one, trans-caryophyllene, and nonanal are important markers (VIP > 1) differentiating samples collected from different floral whorls during the staminate and pistillate phases.

The aromatic profile of *B. variegata* exhibits notable temporal variation across floral whorls. To specifically examine the dynamic change in each floral whorl throughout anthesis, the OPLS-DA method was applied, allowing the identification of candidate VOC markers associated with the staminate and pistillate phases. In petals, 11 VIP-selected compounds were identified across the 2 days of observation. Of these, β-selinene was detected only on the 2nd day, while 1-octanol and β-elemene were exclusive to the 1 st day. Notable differences were also observed in the content of methyl N-hydroxybenzimidate, cadinene, decanal, and β-elemene, all of which were more abundant on the 2nd day (Fig. S1A). For carpels, the VIP score revealed ten VIP-selected compounds. Trans-caryophyllene was exclusively emitted on the 1 st day, while decanal, 2-ethylhexanol, and β-elemene showed increased emission on the 2nd day (Fig. S1B). In stamens, nine VIP-selected compounds belonging to various classes including alcohols, terpenes, aldehydes, and ketones were identified. Decanal and β-elemene were more abundant on the 1 st day, while 2-ethylhexanol showed higher intensity on the 2nd day (Fig. S1C).

Regarding sepals, seven markers were detected (Fig. S1D). 6-methyl-5-hepten-2-one was emitted on the 1 st day, while 2-ethylhexanol was detected only on the 2nd day. VOC content varied markedly on the 2nd day: the content of trans-caryophyllene was approximately 3.2 times lower, while β-elemene levels increased threefold compared to the 1 st day. In trichomes, nine markers were identified (Fig. S1E); β-elemene was exclusive to the 1 st day and 6-methyl-5-hepten-2-one was present only on the 2nd day.

**Fig. 4:**
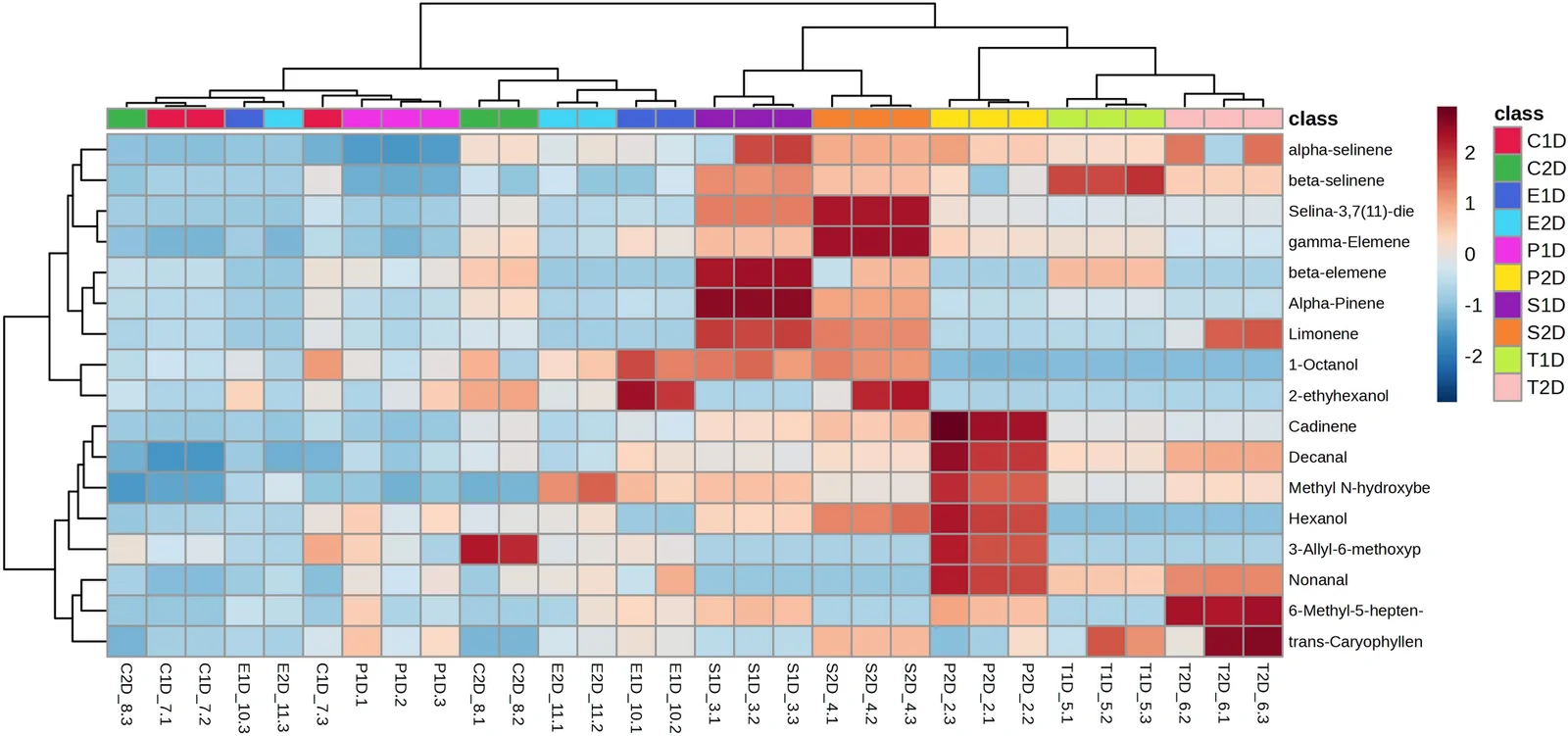
Hierarchical cluster analysis results of floral scent from floral whorls of *Bauhinia variegata*. Analysis restricted to the 17 VOCs detected in more than 50% of samples. Colour scale represents autoscaled abundance (z-score) calculated across samples for each compound. Euclidean distance, Ward’s linkage. *P1D* petals 1 st day, *P2D* petals 2nd day, *C1D* carpels 1 st day, *C2D* carpels 2nd day, *E1D* stamens 1 st day, *E2D* stamens 2nd day, *S1D* sepals 1 st day, *S2D* sepals 2nd day, *T1D* trichomes 1 st day, *T2D* trichomes 2nd day

Hierarchical cluster analysis was performed based on the 17 main VOCs and visualized through a heat map with an associated dendrogram (Fig. 4) to discriminate among floral whorls of *B. variegata* Three main VOC clusters were identified based on abundance patterns across the samples. The first cluster grouped VOCs emitted by stamens and carpels from both days and petals from the 1 st day (P1D). This cluster was characterized by lower overall abundance, except for 2-ethylhexanol and 1-octanol in stamens sampled on the 1 st day. The second cluster grouped sepals from both days (S1D and S2D); β-elemene, selina-3,7(11)-diene, and 2-ethylhexanol showed higher abundance on the 2nd day, while β-pinene was more abundant on the 1 st day. The third cluster grouped VOCs emitted by petals on the 2nd day (P2D) and trichomes on both days. A clear shift in the VOC profile of petals between the 2 days was evident, with higher abundance of cadinene, decanal, hexanol, and nonanal on the 2nd day. In trichomes, β-selinene was abundant on the 1 st day, while 6-methyl-5-hepten-2-one and trans-caryophyllene were more abundant on the 2nd day.

### Floral scent variation across anthesis phases

A comparative analysis of VOCs extracted from different floral whorls of *B. variegata* revealed distinct chemical profiles across the protandrous phases (Table S4). Volatile emissions were dominated by monoterpenes (approximately 85%), particularly α-pinene (55.85 ± 6.08%) and β-pinene (12.91 ± 1.94%), followed by camphene (5.71 ± 0.95%), 3-carene (4.52 ± 0.62%), dodecanol (5.35 ± 1.50%), β-myrcene (2.06 ± 0.22%), limonene (2.54 ± 0.36%), and nonadecane (1.69 ± 0.55%). Although these compounds were present in all phases, their relative abundance varied subtly among the staminate (SP), pistillate (PP), and complete anthesis (CA) phases. The CA phase showed greater chemical heterogeneity, evidenced by higher standard deviations, suggesting dynamic modulation of scent composition during floral longevity.

**Fig. 5:**
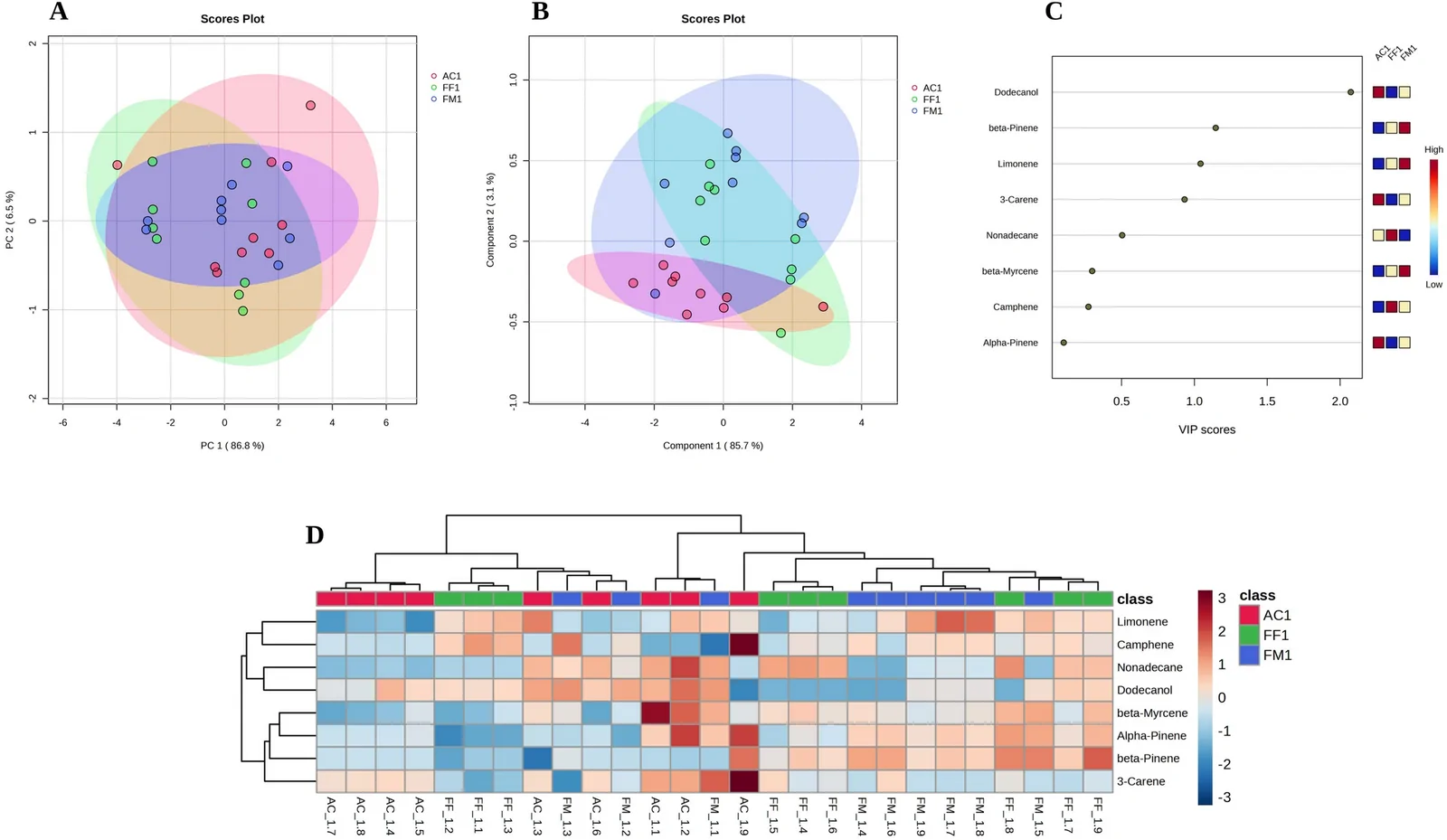
Multivariate analysis of the volatile chemical profile of *Bauhinia variegata* during the anthesis phases (AC1, complete anthesis; FF1, pistillate phase; FM1, staminate phase). **A** Principal component analysis (PCA) score plot (PERMANOVA: F = 1.10, R^2^ = 0.084, p = 0.339). **B** Partial least squares discriminant analysis (PLS-DA) score plot (three components; R^2^ = 0.548, Q^2^ = 0.072; permutation *p* = 0.004). **C** Variable importance in projection (VIP) scores from PLS-DA. **D** Heatmap of the relative abundance of volatile compounds with hierarchical clustering

PCA captured approximately 93% of the total variance, with PC1 and PC2 accounting for 86.8% and 6.5%, respectively (Fig. 5A) (PERMANOVA: *F* = 1.10, R^2^ = 0.084, *p* = 0.339; pairwise comparisons: CA vs PP p-adj = 0.438, CA vs SP p-adj = 0.615, PP vs SP p-adj = 0.548). This high percentage indicates that a few major compounds drive the chemical variation during anthesis. A partial overlap among the three phases was observed, with a tendency toward clustering within each phase. However, considerable heterogeneity, especially between CA and SP samples, was evident in the central region of the plot. This overlap suggests that chemical transitions during anthesis do not follow a strictly linear pattern and are not fully aligned with protandrous phases. PCA loadings showed that dodecanol contributes strongly to PC1, while α-pinene and nonadecane contribute significantly to PC2, indicating that phase differentiation is mainly determined by opposing patterns in aliphatic compounds (dodecanol and nonadecane) and monoterpenes (α-pinene, β-pinene, and limonene).

PLS-DA separated the groups with substantial overlap remaining (three components; R^2^ = 0.548, Q^2^ = 0.072; permutation test *p* = 0.004) (Fig. 5B). The low Q^2^ indicates limited predictive power and the greater chemical heterogeneity of CA, consistent with its transitional status within the protandrous sequence. VIP score values revealed dodecanol (VIP = 2.07), β-pinene (VIP = 1.15), and limonene (VIP = 1.04) as the compounds with the greatest discriminatory power among anthesis phases (Fig. 5C). One-way ANOVA detected no significant difference among phases for any individual compound (all *p* > 0.05; Table S4). Hierarchical cluster analysis (Fig. 5D) revealed complex relationships among samples that extend beyond predefined phenological classifications, with several subclusters containing samples from different phases, reinforcing the dynamic nature of scent composition throughout floral longevity.

### Localization and morphology of cavitated secretory trichomes

**Fig. 6:**
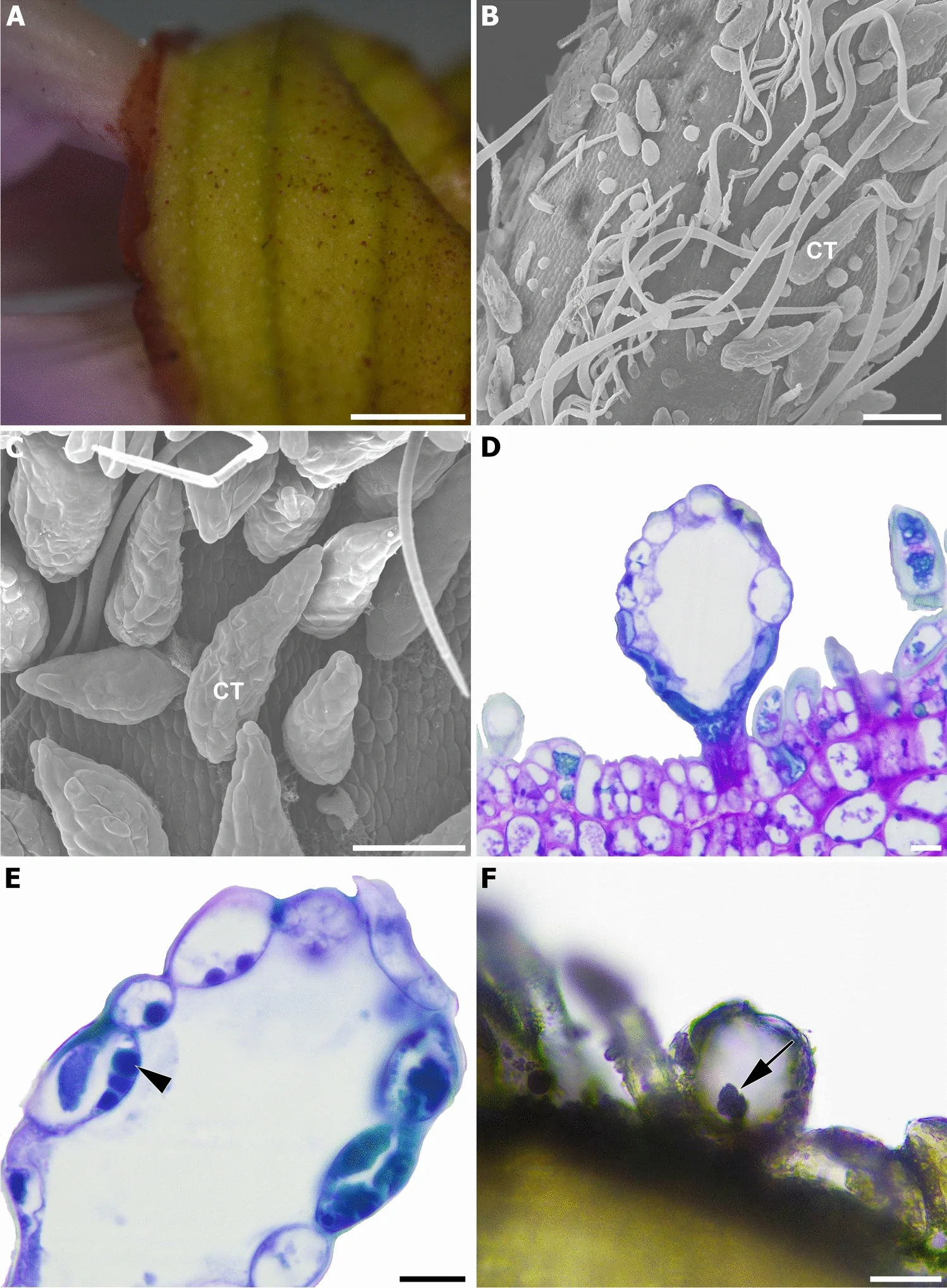
Cavitated secretory trichomes in *Bauhinia variegata* and their exudate. **A** Light micrograph of a calyx cleft margin stained with neutral red, indicating potential sites for volatile compound accumulation. **B-C** Scanning electron micrograph (SEM) of the cavitated secretory trichomes on the style **(B)** and ovary surface **(C)**. **D-F** Light micrographs of cavitated secretory trichomes in cross section on carpels **(D-E)** and on sepal **(F)**, revealing phenolic compounds in their cells, indicated by an arrowhead in **(E)** and internal terpenes droplets in **(F)**, indicated by an arrow. Scale bars: **A** = 2 mm; **B-C; D-F** = 100 µm; **E** = 10 µm

Histochemical analysis with neutral red staining revealed the presence of structures with the potential for volatile compound accumulation in both sepals (Fig. 6A, F) and carpels (Fig. 6B–E) of *B. variegata*, regardless of the anthesis phase. This result suggests that both organs may contribute to the production and emission of VOCs. The positive reaction with Nadi reagent in sepal sections confirmed the presence of terpenes (Fig. 6D–F).

The cavitated secretory trichomes in *B. variegata* from both sepals and carpels have a short, biseriate stalk and are connected to a head formed by a layer of secretory epithelium cells arranged around an oval lumen (Fig. 6D). The epithelium cells of the head produce phenolic compounds and terpenes; the terpenes are released and stored into the lumen (Fig. 6E–F).

### Floral resource: nectar

Nectar production in *B. variegata* flowers varied throughout anthesis. During the first 12 h post-anthesis, corresponding to the first half of the staminate phase, the mean nectar volume per flower was 7.06 ± 4.46 µLᵃ, with a mean sugar concentration of 0.36 ± 0.06 mg/µLᵃ and 2.49 ± 1.53 mgᵃ of total sugar content. During the second half of the staminate phase, nectar volume increased significantly to 12.81 ± 3.99 µLᵇ, whereas sugar concentration remained statistically similar to that recorded during the first half of the staminate phase (0.30 ± 0.09 mg/µLᵃ). Total sugar content increased to 4.11 ± 2.12 mgᵃᵇ but did not differ significantly from either period.

During the following day, corresponding to the pistillate phase, nectar production reached its highest values. In the first 12 h, nectar volume increased to 15.14 ± 5.39 µLᵇ, significantly higher than that recorded during the first 12 h of anthesis and the final 12 h preceding floral senescence. During this period, sugar concentration also increased to 0.34 ± 0.04 mg/µLᵇ, differing significantly from the other periods. Total sugar content reached 5.19 ± 1.98 mgᵇ, significantly higher than that recorded during the first 12 h of anthesis and the final 12 h of the pistillate phase, while the intermediate value observed during the second half of the staminate phase (4.11 ± 2.12 mgᵃᵇ) did not differ significantly from either period.

In the final 12 h preceding floral senescence, nectar volume decreased significantly to 5.35 ± 4.84 µLᵃ, while sugar concentration decreased to 0.28 ± 0.04 mg/µLᵃ and total sugar content to 2.35 ± 1.34 mgᵃ. Overall, nectar production was highest during the first 12 h of the pistillate phase, when both nectar volume and total sugar content reached their maximum values.

### Molecular docking

Molecular docking of eight floral VOCs plus the positive reference ligand eugenol against *Apis mellifera* odourant-binding protein 14 (AmelOBP14; PDB: 3S0D) yielded positive ChemPLP fitness scores for all compounds tested, indicating accommodation within the binding cavity. The complete docking dataset is presented in Table S5. Ranking by top-ranked fitness score was as follows: β-elemene (57.76) > trans-caryophyllene (52.47) > eugenol (49.97) > S-limonene (42.92) > R-limonene (41.72) > α-pinene (40.06) > β-pinene (39.87) > nonanal (39.40) > 6-methyl-5-hepten-2-one (37.82). Among the limonene enantiomers, S-limonene yielded slightly higher ChemPLP values than R-limonene. The docking results were consistent across the generated poses for each ligand, with limited variation among the top-ranked solutions.

Eugenol, included as a positive reference ligand with experimentally characterized binding to AmelOBP14, showed the highest ChemPLP score among the non-sesquiterpenic ligands and a non-zero hydrogen-bond contribution in the top-ranked pose, unlike the floral VOCs analyzed here, whose best-ranked solutions were dominated by nonpolar interactions. For the purposes of focused interpretation, Table 1 summarizes the four ligands emphasized in the present study: eugenol as the positive reference ligand, β-elemene and trans-caryophyllene as the sesquiterpenes with the strongest structural performance in docking, and nonanal as the floral VOC with the clearest biological association with the pistillate phase.

**Table 1:** Comparative docking results for the four ligands emphasized in the present study: eugenol (positive reference), β-elemene, nonanal, and trans-caryophyllene

Ligand	Top-ranked ChemPLP fitness	H-bond (best pose)	Torsion penalty (best pose)	Main interacting residues	Dominant interaction profile
Eugenol	49.97	1.992	0.3893	LEU10, PHE54, VAL71, LEU75, SER108, VAL104, VAL107, LYS111, MET113, VAL116	Mixed profile with hydrogen bonding, π-sulfur, and hydrophobic contacts
β-Elemene	57.76	0.000	0.0920	LEU6, LEU10, ILE50, LEU51, PHE54, ILE56, ILE68, VAL71, MET72, LEU75, VAL104, VAL107, LYS111, MET113, VAL116	Predominantly hydrophobic; broad residue coverage with alkyl and π-alkyl contacts
Nonanal	39.40	0.000	0.3715	LEU10, PHE54, VAL71, LEU75, LYS111, MET113, VAL116	Predominantly hydrophobic; alkyl and π-alkyl contacts
trans-Caryophyllene	52.47	0.000	0.0000	LEU10, ILE50, PHE54, VAL71, MET72, LEU75, VAL104, VAL107, LYS111, MET113, VAL116	Predominantly hydrophobic; broader residue coverage and π-alkyl contact

**Fig. 7:**
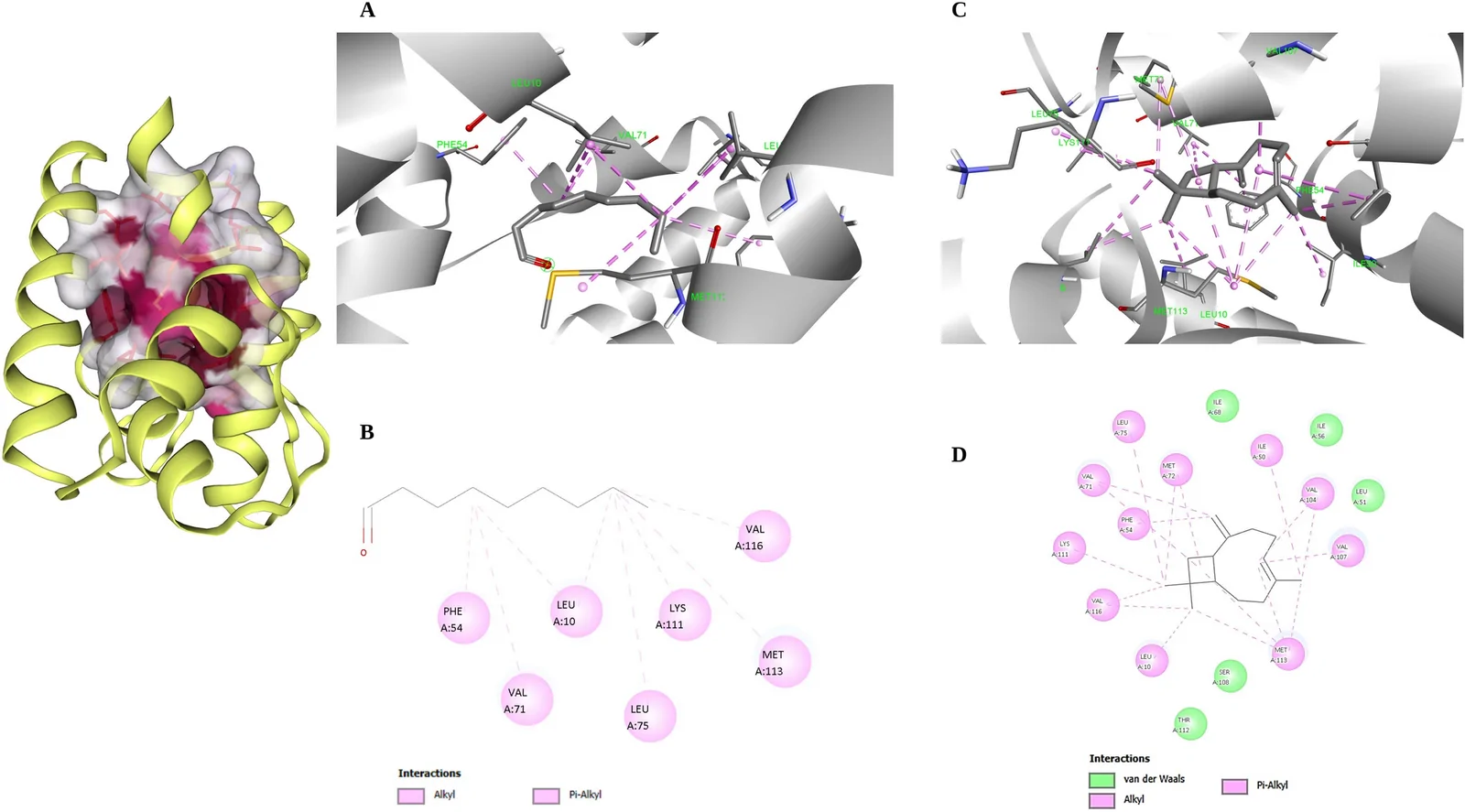
Structural analysis and intermolecular interactions of selected floral VOCs with AmelOBP14 (PDB: 3S0D). Left panel: three-dimensional structure of AmelOBP14 showing the binding cavity identified through structure-based detection. The protein surface is colored to highlight the hydrophobic binding site (pink/magenta regions), with the ribbon representation showing the characteristic α-helical fold of this C-minus odourant-binding protein. **A-B** Nonanal. **A** Three-dimensional view of the nonanal–AmelOBP14 complex within the binding cavity. (B) Two-dimensional diagram of intermolecular interactions showing alkyl contacts with LEU10, VAL71, LEU75, LYS111, MET113, and VAL116, and a p-alkyl interaction with PHE54. **C-D** *trans*-Caryophyllene: **(C)** Three-dimensional conformation of the *trans*-caryophyllene–AmelOBP14 complex. **D** Two-dimensional mapping of non-covalent interactions showing alkyl contacts with LEU10, VAL71, MET72, LEU75, VAL104, VAL107, LYS111, MET113, and VAL116, and p-alkyl interactions with PHE54 and ILE50. In both complexes, all contacts are exclusively hydrophobic (alkyl and p-alkyl), consistent with the apolar character of the AmelOBP14 binding cavity. Interaction types: alkyl (pink), p-alkyl (light pink)

Structural analysis of the top-ranked poses for nonanal and trans-caryophyllene revealed distinct contact networks within the shared hydrophobic cavity of AmelOBP14 (Fig. 7A–D). In the nonanal complex (Fig. 7A, B), the ligand was accommodated along the hydrophobic cavity through alkyl contacts involving LEU10, VAL71, LEU75, LYS111, MET113, and VAL116, together with a π-alkyl interaction with PHE54. The aldehyde carbonyl remained unengaged by hydrogen-bond partners, consistent with the zero H-bond contribution in the docking scores. Small torsional penalties were consistently recorded across the top three poses (0.30–0.37 fitness units), indicating conformational adjustment of the linear chain within the cavity.

By contrast, the trans-caryophyllene complex (Fig. 7C, D) showed a broader hydrophobic contact network. In addition to the residues shared with nonanal (LEU10, PHE54, VAL71, LEU75, LYS111, MET113, and VAL116), trans-caryophyllene engaged ILE50, MET72, VAL104, and VAL107, recruited by its larger bicyclic skeleton. The exocyclic methylene group also contributed a π-alkyl interaction with PHE54. Trans-caryophyllene showed zero torsional penalty across all top-ranked poses, indicating optimal geometric complementarity with the cavity without conformational strain. The broader residue coverage and absence of torsional cost account for its superior ChemPLP score relative to nonanal.

Taken together, these results support the selection of trans-caryophyllene and nonanal for downstream interpretation on complementary grounds: trans-caryophyllene because it combined high docking performance with zero torsional penalty and was advanced to detailed structural and dynamic analyses, and nonanal because it combined structural compatibility with direct biological relevance in the floral dataset, increasing in petals during the pistillate phase.

### Molecular dynamics simulation of AmelOBP14 complexes

**Fig. 8:**
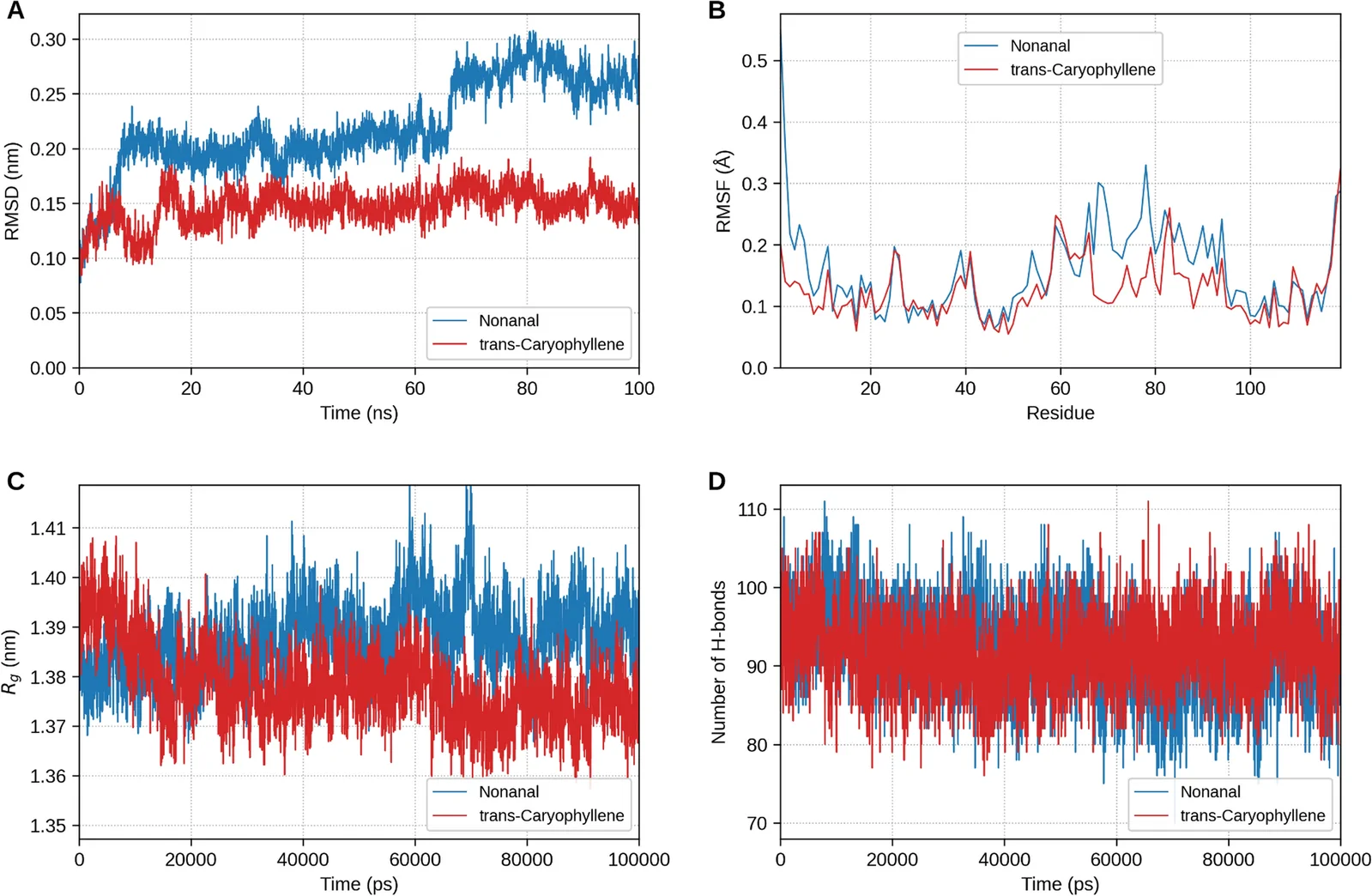
Molecular dynamics simulations of the nonanal–AmelOBP14 and *trans*- caryophyllene–AmelOBP14 complexes (100 ns). **A** Backbone RMSD over time. **B** Per-residue RMSF over the production phase (20–100 ns). **C** Radius of gyration (*R*g) over time. **D** Number of intramolecular hydrogen bonds over time. Blue: nonanal; red: *trans*-caryophyllene

Molecular dynamics simulations were used to compare the global stability of selected AmelOBP14 complexes under dynamic conditions. In the main comparative analysis, the two floral VOCs of greatest biological relevance in the present system—nonanal and trans-caryophyllene—showed distinct dynamic profiles over 100 ns (Fig. 8). The trans-caryophyllene complex displayed the lowest backbone RMSD throughout the trajectory, fluctuating around a stable plateau near 0.15–0.17 nm. By contrast, the nonanal complex showed higher backbone deviation and a mid-trajectory shift between approximately 55 and 65 ns, after which the system stabilized at a second plateau around 0.26–0.28 nm (Fig. 8A). Per-residue RMSF analysis showed that both complexes preserved a generally stable protein framework, with fluctuations concentrated in terminal and loop regions. However, the nonanal complex exhibited higher flexibility than the trans-caryophyllene complex in the N-terminal region and in segments around residues 60–85, including peaks near residues 68 and 78 (Fig. 8B). The trans-caryophyllene complex showed lower overall fluctuation amplitudes across most of the sequence. Radius of gyration values remained broadly stable in both systems, indicating preservation of overall compactness throughout the simulations. Nevertheless, the trans-caryophyllene complex maintained slightly lower Rg values than the nonanal complex across most of the trajectory, consistent with a more compact conformational state (Fig. 8C). The number of intramolecular hydrogen bonds also remained within a similar range in both systems over time, supporting preservation of the global protein framework (Fig. 8D). Eugenol, included as a positive dynamic benchmark, showed an intermediate stability profile relative to nonanal and trans-caryophyllene, consistent with its experimentally validated binding to AmelOBP14; an additional MD analysis of the β-elemene complex also indicated global stability under dynamic conditions (Table S5).

## Discussion

Whether protandry in *B. variegata* L. is expressed solely through sporangiate organ transitions, or whether it is accompanied by coordinated changes in other floral traits, including scent composition and nectar allocation, was the central question examined here. Dichogamous plants must maintain floral visitation across both staminate and pistillate phases even though these phases differ in reproductive role and reward presentation (Lloyd 1984; Aizen et al. 2002), making such coordination ecologically significant. The results show that *B. variegata* expresses a temporally coordinated floral phenotype in which morphology, scent composition, and nectar production all vary across anthesis. This coordination can be described robustly at the levels of floral structure, volatile emission, anatomy of secretory structures, and nectar scheduling. These patterns suggest direct effects on visitor behaviour; however, field and behavioural studies are still required for confirmation.

### Protandry: dynamics of floral display and visual cues across temporal phases

The combination of pollen release during the 1 st day of anthesis, stronger stigma receptivity on the 2nd day, and marked changes in style position confirms a clear protandrous pattern in *B. variegata*. Floral function in this species is therefore sequential rather than simultaneous, with staminate and pistillate phases expressed over a floral lifespan of approximately 2–3 days. This temporal organization, in which reproductive phases are segregated through coordinated morphological and physiological shifts, is consistent with reports for congeneric species such as *B. purpurea* and *B. forficata*, and more broadly with the occurrence of dichogamy in other Cercidoideae taxa (Rao 2020; Neto 2013; Lau et al. 2009).

The most notable of the phase-dependent morphological transitions in *B. variegata* was the transition from a shorter, less conspicuous style in the staminate phase to a longer, curved, pink style in the pistillate phase. This pattern reflects structural reconfiguration of the flower as it shifts from pollen presentation to stigma receptivity. Similar transitions have been documented in other protandrous flowers, such as *Scaevola taccada* (Gaertn.) Roxb. (Goodeniaceae), and *Gentianella saxosa* (G.Forst.) Holub and *G. serotina* (Cockayne) T.N.Ho & S.W.Liu (Gentianaceae), in which the temporal segregation of sexual function is reinforced by changes in the relative position or accessibility of sporangiate organs (Webb and Littleton 1987; Xu et al. 2022). The style movement observed here is therefore best interpreted as part of the developmental and functional expression of protandry.

By contrast, petal reflectance remained essentially unchanged throughout anthesis. This indicates that reproductive phase transition in *B. variegata* is not accompanied by major changes in petal spectral properties. The visual component of the floral display thus appears relatively constant, whereas phase-dependent variation is more evident in sporangiate organs morphology, scent, and nectar, without implying that any single cue alone determines visitor behaviour (Raguso and Weiss 2015; Dötterl and Gershenzon 2023).

### Olfactory cues and temporal dynamics of VOCs

The volatile profile of *B. variegata* was dominated by terpenes, especially α-pinene and β-pinene, but also included aldehydes, alcohols, hydrocarbons, and sesquiterpenes. This composition places the species within the broad chemical spectrum commonly reported for floral bouquets (Dobson and Bergström 2000; Knudsen et al. 2006). More relevant than the diversity of compounds detected, however, is the finding that scent emission in *B. variegata* is spatially structured among floral organs and temporally modulated across anthesis. Current syntheses on floral volatiles emphasize that these compounds often play multiple roles beyond pollinator signalling, including mediation of antagonistic interactions and responses to environmental context (Dötterl and Gershenzon 2023). The data thus demonstrate temporal and organ-specific scent modulation but do not by themselves establish behavioural function, and a conservative reading of the results is warranted.

Multivariate analyses revealed that sepals, petals, stamens, carpels, and cavitated trichomes did not contribute equally to the floral bouquet. *B. variegata* therefore presents an intrafloral scent mosaic, with different floral structures contributing differentially to the overall chemical phenotype. Such spatial heterogeneity has been emphasized in studies showing that floral odour can be partitioned among organs and that close-range floral scent patterns may vary substantially within a single flower (Raguso 2009; Raguso and Weiss 2015; García et al. 2021). In *B. variegata*, this pattern was particularly evident in sepals and carpels, which combined histochemical evidence of terpene-associated secretion with distinct VOC signatures.

Temporal analysis of VOCs further showed that chemical transitions across anthesis were not abrupt. Ordination detected no significant separation among phases (PERMANOVA *p* = 0.339; no pairwise comparison reached significance), and no individual compound differed significantly among them. Although the supervised model separated the three groups, overlap among phases remained evident, especially for complete anthesis. This pattern suggests that scent modulation accompanies the floral transition from staminate to pistillate function, occurring progressively rather than as a sharp shift between phases. The chemical phenotype instead changes progressively through floral longevity, a pattern consistent with the gradual floral trait reconfiguration reported across phases in other protandrous species (Primack 1985; Pellmyr 1986; Dani et al 2021). Differences among floral organs, by contrast, were strongly supported (PERMANOVA R^2^ = 0.953), indicating that spatial partitioning of scent is a stronger organizing principle in *B. variegata* than temporal modulation across anthesis.

Among the compounds identified, nonanal merits closer examination because it increased in petals on the 2nd day of anthesis, when the stigma was receptive and nectar volume was highest. Nonanal is accordingly best interpreted here as a phase-associated candidate volatile, although it is not a demonstrated attractant. Nonanal has also been identified as a scent component specifically associated with honeybee-pollinated flowers, being detected across twelve unrelated species and absent from wind-pollinated relatives (Liu et al. 2022). RNAi silencing of AmelOBP2 in *A. mellifera ligustica* markedly reduces the electroantennogram response of forager bees to 1-nonanal, indicating that this aldehyde is processed by a characterized peripheral olfactory pathway (Zhang et al. 2025); in the present system, nonanal showed structural compatibility with AmelOBP14 and remained bound throughout the 100 ns simulation (see Figs. 7 and  8 ). Studies with bees have shown that some floral compounds can elicit electrophysiological responses without necessarily producing positive behavioural responses in olfactometer assays (Ma et al. 2021; Zhang et al. 2022). Thus, the temporal association observed here is biologically suggestive, but it does not substitute for behavioural validation.

### Cavitated secretory trichomes and floral secretory architecture

The histochemical and anatomical results reinforce the interpretation that floral scent-related secretion in *B. variegata* is not restricted to a single organ. Neutral red staining and the positive Nadi reaction indicate metabolically active secretory regions and terpene-associated secretion in sepals and carpels, while microscopy confirmed the occurrence of cavitated secretory trichomes with a lumen capable of storing secreted substances. These observations are consistent with previous studies in *Bauhinia* sensu stricto describing cavitated secretory trichomes as unusual secretory structures involved in the production and storage of terpenoid and phenolic compounds (Duarte-Almeida et al. 2015; Marinho et al. 2016; Silva et al. 2024).

The VOC profiles of trichomes also varied between the first and 2nd day of anthesis, indicating that these structures are chemically dynamic rather than static. trans-caryophyllene and 6-methyl-5-hepten-2-one are widespread constituents of angiosperm floral scent; each recorded in more than half of the families surveyed by Knudsen et al. (2006). Their increase in trichomes on the 2nd day is consistent with the idea that trichomes contribute to the temporal structuring of the floral bouquet. However, the ecological role of these changes remains open. The data thus support the conclusion that cavitated trichomes participate in the production of secretions underlying the floral scent phenotype of *B. variegata*.

### Nectar dynamics and temporal reward presentation

Nectar production also varied substantially across anthesis. Volume increased from the early staminate phase to the first half of the pistillate phase, then declined as flowers approached senescence, whereas sugar concentration varied more modestly. The peak in nectar volume and total sugar content during the first 12 h of the pistillate phase coincided with the period of strongest stigma receptivity. Stigma receptivity was scored qualitatively by peroxidase activity, so this temporal correspondence is descriptive and was not tested statistically. It nonetheless indicates that nectar presentation in *B. variegata* is phase-dependent, consistent with patterns reported in previously studied models (Pyke 1978a, b, c; Cruden and Hermann 1983).

### Molecular docking and molecular dynamics as complementary mechanistic support

Molecular docking and molecular dynamics simulations were used here as complementary approaches to assess whether selected floral volatiles of *B. variegata* are structurally compatible with a characterized bee odourant-binding protein and whether the protein framework remains stable under dynamic simulation conditions in the presence of these ligands. The receptor used was AmelOBP14 from *Apis mellifera* (PDB ID: 3S0D; Spinelli et al. 2012), and eugenol was included as a positive reference ligand because its sub-micromolar dissociation constant for this OBP has been experimentally determined (Spinelli et al. 2012; Mayack et al. 2023).

All tested ligands showed positive ChemPLP scores in docking analyses, with β-elemene yielding the highest top-ranked value, followed by trans-caryophyllene, eugenol, and nonanal. The interaction profiles of trans-caryophyllene and nonanal were dominated by hydrophobic contacts, consistent with the apolar character of the AmelOBP14 cavity (Spinelli et al. 2012). These docking results indicate that phase-associated floral VOCs of *B. variegata* are structurally compatible with a bee odourant-binding protein, but they do not by themselves establish attraction, receptor specificity, or behavioural preference. 

The MD simulations refine this interpretation. Trans-caryophyllene and nonanal both maintained globally stable protein frameworks over 100 ns, but their dynamic behaviour differed. The trans-caryophyllene complex showed lower backbone RMSD, lower residue-level fluctuation, and slightly greater compactness than the nonanal complex, indicating a more rigid structural state of the protein under this ligand condition. The nonanal complex remained globally stable, although it showed a mid-trajectory conformational transition and greater flexibility in loop regions flanking the cavity entrance. The MD data are therefore best interpreted as evidence that the protein remains structurally stable in the presence of both ligands, with trans-caryophyllene associated with the more rigid framework and nonanal with a more flexible one. An additional MD analysis of the β-elemene complex also indicated a stable protein framework under dynamic conditions, supporting its compatibility with the same hydrophobic cavity.

These differences are biologically informative because the compounds occupy different positions in the floral phenotype of *B. variegata*. Trans-caryophyllene showed strong structural compatibility in docking and the most stable dynamic profile, while nonanal showed the clearest temporal association with the pistillate phase, increasing in petals when stigma receptivity and nectar volume were highest. Thus, the focal ligands contribute in distinct ways to the interpretation of the floral chemical phenotype: trans-caryophyllene as a focal sesquiterpene with strong structural compatibility and the most stable dynamic profile, nonanal as the ligand with the strongest integration between floral phase association and mechanistic olfactory plausibility, and β-elemene as additional support that the AmelOBP14 cavity is compatible with sesquiterpenic floral signals.

Under a conservative interpretation, the computational analyses show that selected phase-associated VOCs of *B. variegata* are structurally compatible with a bee OBP and that the protein framework remains stable in the presence of the ligands tested under MD conditions. This supports mechanistic plausibility of peripheral olfactory detectability, while electrophysiological and behavioural assays remain necessary for ecological validation.

## Conclusions

The results indicate that *Bauhinia variegata* L. expresses protandry through a coordinated sequence of floral changes involving sporangiate organs position, stigma receptivity, scent composition, secretory activity, and nectar presentation. Petal reflectance remains stable throughout anthesis, whereas floral chemistry and nectar availability vary through time, particularly between the first and 2nd day of anthesis. Cavitated secretory trichomes and sepals contribute to the secretory framework underlying this phenotype, and the docking analyses further show that key floral volatiles are structurally compatible with a bee odourant-binding protein.

Under a conservative interpretation, these findings do not establish how pollinators behaviourally respond to the phase-dependent floral phenotype of *B. variegata*. They do, however, demonstrate that this species displays a temporally organized floral system in which different trait classes are reconfigured across anthesis. The study therefore provides a descriptive and mechanistic basis for future work integrating floral chemistry with direct visitor observations, electrophysiology, and behavioural assays.

## Supplementary Information

Below is the link to the electronic supplementary material.

Supplementary file1 (PDF 601 KB)

## Data Availability

All the data generated or analysed during this study are included in this published article and its Supplementary Information.
