# Supplementary material for: Floral dynamics in the protandrous flowers of Bauhinia variegata (Fabaceae): phase-specific morphology, scent, and nectar production
Source: Planta. 2026 Sep 19;264(5):135. doi: 10.1007/s00425-026-05146-0 (PMC13589757; doi:10.1007/s00425-026-05146-0)
Supplement: Supplementary file 1 [file 425_2026_5146_MOESM1_ESM.pdf]

Supporting Information

PLANTA

Floral dynamics in the protandrous flowers of *Bauhinia variegata* (Fabaceae): phase-specific morphology, scent, and nectar production

Andrews Vinicius Santos da Silva[1], Vidal de Freitas Mansano[1,2], Ana Claudia Fernandes Amaral[3], Jefferson Diocesano da Cruz[3], Fabrício de Oliveira Silva[4], Dalton Guimarães Veloso[5], Walter Sotto Mayor Fernandes Neto[6], Jefferson Rocha de Andrade Silva[6], Pedro Joaquim Bergamo[7,8], Leandro Freitas[2,8], Juliana Villela Paulino[1,4]

[1]*Programa de Pós-Graduação em Botânica, Escola Nacional de Botânica Tropical, Instituto de Pesquisas Jardim Botânico do Rio de Janeiro, Rua Pacheco Leão 2040, Rio de Janeiro, RJ, 22460-036, Brazil.*
[2]*Instituto de Pesquisas Jardim Botânico do Rio de Janeiro, DIPEQ, Rua Pacheco Leão 915, Rio de Janeiro, RJ, 22460-030, Brazil.*
[3]*Laboratório de Plantas Medicinais e Derivados, Farmanguinhos, Fundação Oswaldo Cruz, Rio de Janeiro, RJ, 21041-250, Brazil.*
[4]*Departamento de Produtos Naturais e Alimentos, Faculdade de Farmácia, Centro de Ciências da Saúde, Universidade Federal do Rio de Janeiro (UFRJ), Rio de Janeiro, RJ, 21941-902, Brazil.*
[5]*Programa de Pós-Graduação em Ciências Farmacêuticas, Faculdade de Farmácia, Centro de Ciências da Saúde, Universidade Federal do Rio de Janeiro (UFRJ), Rio de Janeiro, RJ, 21949-900, Brazil.*
[6]*Laboratório de Cromatografia, Departamento de Química, Instituto de Ciências Exatas, Universidade Federal do Amazonas (UFAM), Manaus, AM, 69077-000, Brazil.*
[7]*Instituto de Biociências, Universidade Estadual Paulista (UNESP), Câmpus de Rio Claro, Avenida 24 A, 1515, Rio Claro, SP, 13506-900, Brazil.*
[8]*Instituto Nacional de Ciência e Tecnologia em Polinização (INPol), Instituto de Pesquisas Jardim Botânico do Rio de Janeiro, DIPEQ, Rua Pacheco Leão 915, Rio de Janeiro, RJ, 22460-030, Brazil.*

Corresponding authors: *jvillelapaulino@yahoo.com.br / Orcid-ID:0000-0003-0072-639X; jrocha_01@yahoo.com.br / Orcid-ID:0000-0003-2569-1306*

Table S1. The raw data for nectar volume, ºBrix, sugar concentration, and total sugar content in Bauhinia variegata flowers during the staminate and pistillate phases.

Staminate phase										Pistilate phase									
00-12 hours					12-24 hours					24-36 hours					36-48 hours				
Flowers	Volume (µl)	°Brix	Concentration (mg/µl)	Total Sugar (mg)	Flowers	Volume (µl)	°Brix	Concentration (mg/µl)	Total Sugar (mg)	Flowers	Volume (µl)	°Brix	Concentration (mg/µl)	Total Sugar (mg)	Flowers	Volume (µl)	°Brix	Concentration (mg/µl)	Total Sugar (mg)
1	2	32	0,362	0,724	27	9,5	29	0,323	3,070	43	11	30	0,336	3,696	57	6	25	0,273	1,638
2	1,6	28	0,310	0,496	28	9	22	0,236	2,130	44	14	35	0,401	5,626	58	2	21	0,224	0,449
3	4	38	0,442	1,771	29	20	31	0,348	6,978	45	16	34	0,388	6,215	59	12,5	27	0,297	3,723
4	1,6	39	0,456	0,730	30	7,5	25	0,273	2,048	46	18	28	0,310	5,588	60	9	20	0,213	1,9175
5	4,6	39	0,456	2,100	31	14	25	0,273	3,823	47	15,5	26	0,285	4,424	61	6,5	27	0,297	1,936
6	1,5	28	0,310	0,465	32	13,5	34	0,388	5,244	48	16,5	30	0,336	5,544	62	15	27	0,297	4,468
7	6,7	39	0,456	3,059	33	18	36	0,415	7,477	49	6,5	30	0,336	2,184	63	7	30	0,336	2,352
8	7,1	41	0,484	3,441	34	18	29	0,323	5,817	50	19,5	29	0,323	6,302					
9	3,48	35,6	0,409	1,426	35	12,5	22	0,236	2,958	51	3	28	0,310	0,931					
10	2,5	26	0,285	0,713	36	7,5	20	0,213	1,597	52	20	34	0,388	7,769					
11	6	21	0,224	1,348	37	14,5	43	0,513	7,443	53	13,5	26	0,285	3,853					
12	12	29	0,323	3,878	38	14,5	22	0,236	3,432	54	23	30	0,336	7,728					
13	11	34	0,388	4,273	39	8	21	0,224	1,798	55	16	33	0,375	6,002					
14	7,5	31	0,348	2,617	40	13	25	0,273	3,549	56	19,5	31	0,3489	6,804					
15	4,5	27	0,297	1,340	41	16	35	0,401	6,429										
16	8	26	0,285	2,283	42	9,5	20	0,213	2,024										
17	13,5	29	0,323	4,363															
18	18	30	0,336	6,048															
19	7	34	0,388	2,719															
20	16	32	0,362	5,792															
21	12,5	28	0,310	3,881															
22	5,5	31	0,348	1,919															
23	9	30	0,336	3,024															
24	5,5	31	0,348	1,919															
25	7,5	30	0,336	2,520															
26	5	32	0,362	1,810															
**Mean**	**7,060**	**31,561**	**0,357**	**2,487**		**12,812**	**27,437**	**0,305**	**4,114**		**15,142**	**30,285**	**0,340**	**5,190**		**6,805**	**25,285**	**0,277**	**2,355**
**Standard deviation**	**4,461**	**4,812**	**0,063**	**1,532**		**3,995**	**6,791**	**0,087**	**2,124**		**5,393**	**2,867**	**0,037**	**1,978**		**4,772**	**3,592**	**0,044**	**1,344**

Table S2. Summary of molecular dynamics metrics for apo AmelOBP14 and selected receptor–ligand complexes over 100 ns. Apo values were summarized from the stabilized 80–100 ns interval of the ligand-free trajectory, whereas ligand-bound systems were summarized from the 20–100 ns production interval. RMSD, radius of gyration (Rg), intramolecular hydrogen bonds, solvent-accessible surface area (SASA), and molecular volume are reported as mean ± standard deviation. Qualitative dynamic interpretation is comparative and intended only as conservative mechanistic support.

System name	Simulation length (ns)	Backbone RMSD (mean ± SD, nm)	RMSD plateau range (nm)	Per-residue RMSF mean / key peaks	Radius of gyration (mean ± SD, nm)	No. intramolecular H-bonds (mean ± SD)	SASA (mean ± SD, nm^2)	Volume (mean ± SD, nm^3)	Qualitative dynamic interpretation	Notes
**Apo AmelOBP14**	100	0.233 ± 0.010	0.20–0.27	Mean 0.155; peaks at 1–3, 59, 83, 92–94, 118–119	1.372 ± 0.006	92.3 ± 4.7	69.47 ± 1.12	25.38 ± 0.29	Stable / baseline	Values summarized from the stabilized 80–100 ns interval; terminal and loop regions account for most intrinsic flexibility
**Eugenol–AmelOBP14**	100	0.216 ± 0.016	0.18–0.26	Mean 0.153; peaks at 59, 78, 82, 89–94, 117–119	1.379 ± 0.007	92.4 ± 5.3	69.54 ± 1.47	25.47 ± 0.30	Intermediate / stable	Stable global framework over 20–100 ns; intermediate profile relative to nonanal and trans-caryophyllene
**Nonanal–AmelOBP14**	100	0.230 ± 0.035	0.26–0.28	Mean 0.158; peaks at 1–2, 66, 68–70, 77–78, 82, 94, 118–119	1.389 ± 0.007	91.1 ± 5.4	71.92 ± 1.49	25.68 ± 0.30	Flexible / stable	Global framework preserved, but with a mid-trajectory conformational transition and a second plateau in the final stage
**trans-Caryophyllene–AmelOBP14**	100	0.150 ± 0.012	0.15–0.17	Mean 0.126; peaks at 59–61, 66, 82–83, 118–119	1.376 ± 0.006	91.7 ± 4.8	71.05 ± 1.26	25.59 ± 0.29	Rigid / stable	Most stable complex among the focal floral VOCs; compact trajectory over 20–100 ns
**β-Elemene–AmelOBP14**	100	0.169 ± 0.014	0.16–0.18	Mean 0.128; peaks at 59–61, 66–68, 78–79, 83, 94, 118–119	1.377 ± 0.007	91.8 ± 5.0	70.78 ± 1.35	25.52 ± 0.29	Rigid / stable	Stable trajectory over 20–100 ns; dynamic profile close to trans-caryophyllene

Table S3. Volatile compounds emitted by *Bauhinia variegata* L. floral parts by SPME-headspace-GC-MS (expressed in ppb).

Compound	P1D	P2D	S1D	S2D	T1D	T2D	C1D	C2D	E1D	E2D
Hexanol	53.84 ± 4.76	44.07 ± 1.19	108.25 ± 3.10	25.07 ± 2.58	–	–	25.06 ± 1.40	12.44 ± 1.08	243.62 ± 1.20	316.10 ± 1.21
Methyl N-hydroxybenzimidate	109.22 ± 4.49	500.64 ± 1.47	196.06 ± 3.14	112.15 ± 0.77	94.62 ± 2.94	137.53 ± 1.83	75.10 ± 1.25	24.28 ± 1.41	507.89 ± 1.37	1490.97 ± 2.60
Tricyclene	24.94 ± 2.47	23.20 ± 1.08	–	174.22 ± 1.36	–	–	52.87 ± 1.53	58.22 ± 1.99	–	–
Alpha-Pinene	138.47 ± 3.59	34.75 ± 1.38	1364.54 ± 3.78	425.98 ± 1.64	67.30 ± 3.95	30.70 ± 0.69	375.95 ± 1.70	372.48 ± 1.45	80.37 ± 1.27	59.00 ± 0.54
Camphene	–	–	–	224.47 ± 3.53	7.95 ± 1.42	–	48.40 ± 1.40	44.79 ± 1.69	–	–
6-Methyl-5-hepten-2-one	9.84 ± 1.29	30.45 ± 1.77	22.72 ± 1.87	–	–	9.20 ± 0.42	–	–	35.27 ± 1.93	39.70 ± 1.28
beta-pinene	–	–	446.80 ± 2.95	176.49 ± 1.89	15.40 ± 2.69	–	151.40 ± 1.94	71.62 ± 1.35	–	–
endo-borneol	–	–	–	–	8.45 ± 0.90	21.54 ± 0.51	3.71 ± 0.84	34.64 ± 1.26	–	–
2-ethyhexanol	37.54 ± 2.86	–	–	34.56 ± 2.66	–	–	13.31 ± 0.39	28.98 ± 1.64	88.94 ± 2.39	155.05 ± 4.32
1-Octanol	36.39 ± 5.18	–	22.08 ± 3.67	20.29 ± 1.65	–	–	6.83 ± 0.60	4.59 ± 0.87	70.10 ± 1.78	47.44 ± 1.08
Phenethyl alcohol	–	–	1146.65 ± 2.81	433.66 ± 2.76	–	–	23.64 ± 1.43	–	–	–
Methyl 2-phenylacetate	–	–	24.41 ± 1.73	67.83 ± 1.18	–	–	–	–	–	–
Phenethyl acetate	–	–	93.51 ± 1.61	89.30 ± 3.57	–	–	–	–	–	–
Nonanal	53.23 ± 1.43	83.52 ± 2.22	–	–	21.37 ± 1.16	41.32 ± 0.79	–	19.45 ± 0.92	93.32 ± 1.18	50.79 ± 0.98
Tridecanal	4.13 ± 0.60	7.65 ± 1.23	–	–	4.95 ± 0.84	–	–	–	–	–
Decanal	53.54 ± 1.92	109.62 ± 0.41	19.61 ± 1.25	26.65 ± 0.35	26.50 ± 1.84	43.05 ± 0.75	8.15 ± 0.84	39.20 ± 1.48	71.43 ± 0.65	43.94 ± 1.21
Undecanal	–	10.59 ± 1.77	–	–	–	–	–	–	–	–
GERANIOL	–	25.27 ± 2.92	–	–	–	–	–	–	–	–
3-Allyl-6-methoxyphenol	37.40 ± 0.97	30.06 ± 0.79	–	–	–	–	4.28 ± 0.60	7.19 ± 0.88	120.78 ± 1.32	162.75 ± 1.93
Limonene	11.55 ± 2.22	–	81.88 ± 3.08	49.76 ± 3.04	–	6.59 ± 0.19	25.53 ± 0.34	11.72 ± 0.93	–	–
Tetradecane	–	9.19 ± 0.44	–	–	–	–	–	–	–	–
alpha-cubebene	–	–	–	–	–	–	6.72 ± 1.13	1.46 ± 0.33	–	–
Copaene	5.09 ± 0.93	–	20.73 ± 1.14	–	–	3.02 ± 0.43	7.98 ± 0.82	3.80 ± 0.58	–	–
beta-elemene	28.02 ± 0.97	–	68.54 ± 1.98	176.53 ± 2.39	16.54 ± 0.50	–	22.84 ± 0.93	30.91 ± 1.66	–	–
trans-Caryophyllene	5.55 ± 1.67	12.37 ± 0.87	18.14 ± 1.41	170.57 ± 3.94	2.98 ± 0.77	6.48 ± 0.16	15.17 ± 0.52	–	18.34 ± 0.66	19.46 ± 0.43

Compound	P1D	P2D	S1D	S2D	T1D	T2D	C1D	C2D	E1D	E2D
Geranylacetone	–	40.81 ± 2.23	–	–	–	–	–	–	–	–
gamma-Elemene	163.22 ± 1.19	247.15 ± 2.56	314.06 ± 2.65	970.73 ± 5.97	185.23 ± 1.27	99.94 ± 1.85	260.67 ± 0.94	457.99 ± 1.73	520.46 ± 1.33	313.01 ± 1.96
Octadecanal	–	–	–	–	–	–	–	–	15.83 ± 1.57	–
Farnesol	–	–	–	–	9.29 ± 0.74	139.00 ± 0.81	–	–	–	–
beta-selinene	–	11.52 ± 1.72	33.54 ± 1.40	208.47 ± 2.14	5.55 ± 0.50	16.75 ± 0.34	31.39 ± 1.13	13.68 ± 1.96	18.57 ± 0.52	28.60 ± 1.94
alpha-selinene	–	23.76 ± 2.40	46.38 ± 1.33	245.24 ± 3.04	14.09 ± 0.66	35.11 ± 0.56	26.57 ± 0.81	29.52 ± 1.36	23.29 ± 0.63	43.23 ± 0.43
Farnesene	–	179.41 ± 3.20	–	–	–	–	–	–	–	–
alpha-humulene	5.13 ± 0.62	–	–	46.72 ± 5.23	–	–	11.89 ± 0.57	22.88 ± 0.98	–	–
alpha-guaiene	–	–	–	121.16 ± 3.87	–	–	11.44 ± 0.56	4.58 ± 1.26	–	–
NEOALLOOCIMENE	–	–	–	–	–	–	14.95 ± 1.77	15.61 ± 0.29	–	–
delta-guaiene	–	–	–	24.03 ± 3.62	–	–	3.29 ± 0.94	–	–	–
Cadinene	11.58 ± 1.52	245.69 ± 3.41	36.21 ± 2.78	51.97 ± 4.52	20.74 ± 0.87	15.83 ± 0.33	31.02 ± 1.16	44.49 ± 0.70	42.24 ± 0.41	29.88 ± 1.61
gamma-selinene	–	–	–	–	–	–	26.85 ± 0.53	40.96 ± 0.93	–	–
Selina-3,7(11)-diene	13.43 ± 1.01	23.38 ± 1.83	93.95 ± 0.15	184.64 ± 2.45	14.57 ± 1.01	14.63 ± 0.24	37.62 ± 1.94	43.68 ± 0.45	19.56 ± 0.40	23.71 ± 1.20
Cadalene	–	–	–	–	9.54 ± 0.31	–	–	–	–	–
Germacrene B	14.01 ± 0.81	24.04 ± 2.78	–	94.26 ± 2.75	–	–	23.45 ± 0.70	45.83 ± 0.10	–	–
beta-Panasinsene	–	–	–	175.33 ± 3.26	–	–	–	–	–	–
Dihydro methyl jasmonate	–	11.16 ± 0.24	–	–	–	–	–	–	–	–
Dibutyl adipate	34.18 ± 0.38	3.96 ± 0.46	–	–	–	–	–	–	–	–
Hexadecyl octanoate	165.50 ± 0.76	40.82 ± 3.45	–	–	–	–	–	–	–	–
Isopropyl hexadecanoate	73.95 ± 0.39	64.15 ± 2.19	–	–	–	–	–	–	42.88 ± 2.92	42.11 ± 0.53
Palmitic acid	–	–	–	–	–	–	–	–	19.90 ± 0.70	–
Dihydro methyl jasmonate.1	–	–	–	–	–	–	–	–	19.47 ± 1.95	37.24 ± 1.39
Dotriacontane	–	–	–	–	–	–	–	–	155.26 ± 0.97	66.49 ± 0.43

Values are mean ± standard deviation of three biological replicates per floral organ, each consisting of a pool of flowers collected from a distinct individual tree. – : not detected. P1D: Petals first day; P2D: Petals second day; C1D: Carpels first day; C2D: Carpels second day; E1D: Stamens first day; E2D: Stamens second day; S1D: Sepals first day; S2D: Sepals second day; T1D: Trichomes first day; T2D: Trichomes second day.

Table S4. Composition of volatile organic compounds (VOCs) in the floral scent emissions of *Bauhinia variegata* L. during both protandrous phases, expressed in ppb (mean ± SD). One-way ANOVA per compound across anthesis phases (n = 3 biological replicates per phase).

**Compounds**	**CA**	**PP**	**SP**	**F(2,6)**	**p**
α-Pinene	53.39 ± 11.11	58.05 ± 2.07	56.11 ± 5.06	0.222	0.807
3-Carene	4.56 ± 1.07	4.72 ± 0.42	4.29 ± 0.37	0.225	0.805
Camphene	5.23 ± 1.41	6.33 ± 0.80	5.58 ± 0.63	0.757	0.509
β-Pinene	11.34 ± 3.31	14.15 ± 0.67	13.25 ± 1.85	0.884	0.461
β-Myrcene	1.83 ± 0.27	2.23 ± 0.21	2.13 ± 0.19	4.378	0.067
Limonene	2.08 ± 0.29	2.83 ± 0.43	2.70 ± 0.37	3.024	0.124
Dodecanol	5.27 ± 2.03	5.62 ± 0.53	5.15 ± 1.95	0.101	0.906
Nonadecane	1.47 ± 0.53	2.22 ± 0.67	1.39 ± 0.46	1.743	0.253

CA = Complete Anthesis; PP = Pistillate Phase; SP = Staminate Phase. $F(2,6)$ and p from one-way ANOVA comparing the three anthesis phases (n = 3 biological replicates per phase). No compound differed significantly among phases (all $p > 0.05$); *β*-myrcene approached significance ($p = 0.067$).

Table S5. Molecular docking results for the top-ranked ChemPLP pose of each compound tested against AmelOBP14 (PDB: 3S0D; GOLD Suite v.2024.3.0, ChemPLP scoring function).

Compound	Top-ranked ChemPLP Fitness	H-bond (best pose)	Torsion penalty (best pose)	Nonpolar component (best pose)	Nonpolar (%)
**β-Elemene**	57.76	0.000	0.0920	56.69	98.1
**trans-Caryophyllene**	52.47	0.000	0.0000	51.49	98.1
**Eugenol***	49.97	1.992	0.3893	39.38	78.8
**S-Limonene**	42.92	0.000	0.0142	42.60	99.3
**R-Limonene**	41.72	0.000	0.0199	40.85	97.9
**α-Pinene**	40.06	0.000	0.0000	39.13	97.7
**β-Pinene†**	39.87	0.000	0.0000	39.92	100.1
**Nonanal**	39.40	0.000	0.3715	39.20	99.5
**6-Methyl-5-hepten-2-one**	37.82	0.000	0.3496	36.98	97.8

* **Eugenol**: positive reference ligand with experimentally determined sub-micromolar affinity for AmelOBP14. Its non-zero hydrogen-bond contribution and lower nonpolar fraction distinguish it from the purely hydrophobic floral VOCs.

† **β-Pinene**: the nonpolar percentage slightly exceeding 100% reflects score decomposition and rounding/correction terms in the ChemPLP output.

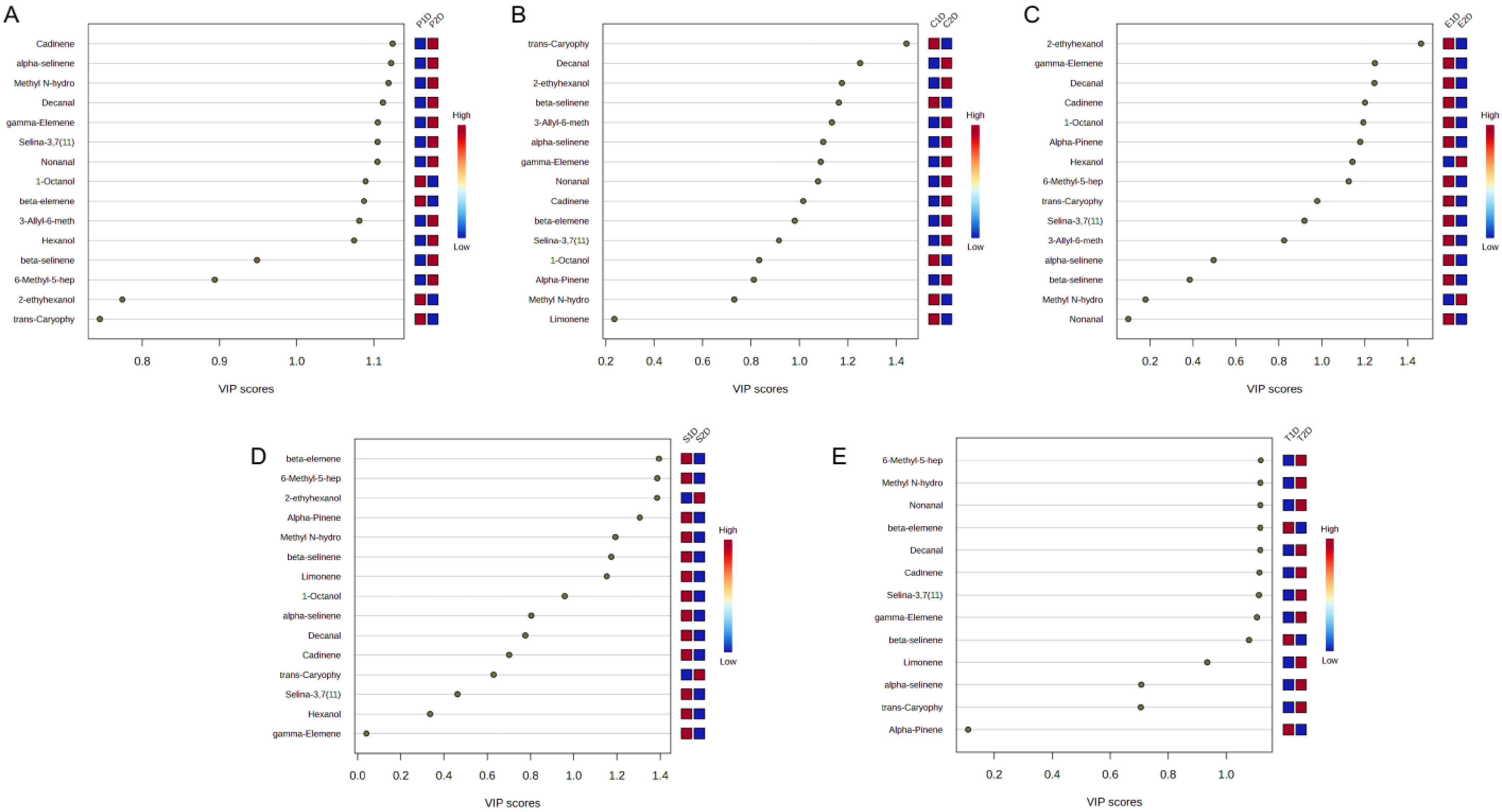

Figure S1: Variable Importance in Projection (VIP) scores from the PLS-DA model of floral-whorl volatile profiles (four components; $R^2 = 0.895$, $Q^2 = 0.793$; permutation test $p < 0.01$; see Fig. 3), shown for the different floral whorls of *Bauhinia variegata* collected on the first and second days. Compounds with VIP > 1.0 are considered discriminatory. P1D: petals, first day; P2D: petals, second day; C1D: carpels, first day; C2D: carpels, second day; E1D: stamens, first day; E2D: stamens, second day; S1D: sepals, first day; S2D: sepals, second day; T1D: trichomes, first day; T2D: trichomes, second day.
